# Predicting individual task contrasts from resting-state functional connectivity using a surface-based convolutional network

**DOI:** 10.1016/j.neuroimage.2021.118849

**Published:** 2021-12-26

**Authors:** Gia H. Ngo, Meenakshi Khosla, Keith Jamison, Amy Kuceyeski, Mert R. Sabuncu

**Affiliations:** a School of Electrical & Computer Engineering, Cornell University and Cornell Tech, United States; b Radiology, Weill Cornell Medicine, United States

**Keywords:** Surface-based convolutional neural network, Task-evoked contrasts, Resting-state functional connectivity

## Abstract

Task-based and resting-state represent the two most common experimental paradigms of functional neuroimaging. While resting-state offers a flexible and scalable approach for characterizing brain function, task-based techniques provide superior localization. In this paper, we build on recent deep learning methods to create a model that predicts task-based contrast maps from resting-state fMRI scans. Specifically, we propose BrainSurfCNN, a surface-based fully-convolutional neural network model that works with a representation of the brain’s cortical sheet BrainSurfCNN achieves exceptional predictive accuracy on independent test data from the Human Connectome Project, which is on par with the repeat reliability of the measured subject-level contrast maps. Conversely, our analyses reveal that a previously published benchmark is no better than group-average contrast maps. Finally we demonstrate that BrainSurfCNN can generalize remarkably well to novel domains with limited training data.

## 1. Introduction

Task-based functional magnetic resonance imaging (tfMRI) has been an indispensable tool for probing neural correlates supporting cognitive, emotional and movement-related processes in the human brain. Activation patterns extracted from tfMRI have been used to characterize the functional anatomy of the human brain (Barch et al., 2013; Besle et al., 2013; Gordon et al., 2017), or derive neural biomarkers for individual behavioral measures such as working memory capacity (McNab and Klingberg, 2008), visual attention (Mukai et al., 2007), loss aversion (Tom et al., 2007) or reading ability (Nijhof and Willems, 2015; Wang et al., 2019). However, tfMRI requires careful design and expensive subject training to elicit the appropriate cognitive components that the experiment intends to investigate (Church et al., 2010; Rosazza et al., 2018). On the other hand, resting-state fMRI (rsfMRI), which measures spontaneous, slow-changing fluctuations of brain activity in the absence of external stimuli has become the workhorse in a growing number of neuroscience studies, in part due to its ease of acquisition and higher tolerance to confounds (Dubois and Adolphs, 2016; Power et al., 2014). RsfMRI can reveal a wide range of large-scale brain networks and states associated with heterogenous cognitive processes (Power et al., 2011; Smith et al., 2009; Yeo et al., 2011). The rich repertoire of information embedded in the intrinsically-generated spontaneous brain activity captured by rsfMRI has also been suggested to be highly correlated with behavioral traits (He et al., 2020; Liégeois et al., 2019) and psychological risk factors (Cherkassky et al., 2006; Tian et al., 2006; Venkataraman et al., 2012), and highly specific to individuals Biswal et al. (2010), Finn et al. (2015) and Amico and Goñi (2018). In this work, we limit our focus to the question of whether task subject-specific contrast maps derived from tfMRI are predictable based on rsfMRI, in healthy subjects. Despite the differences in methodologies, signals captured by tfMRI and rsfMRI are likely to arise from similar anatomical connections and neural processes (Varoquaux and Craddock, 2013), as evidenced by significant overlaps between these two modalities (Biswal et al., 1995; Smith et al., 2009). This suggests that individual task-based brain activity may be predictable from resting-state functional connectivity; indeed, such predictive models based on linear regression have previously been presented (Cole et al., 2016; Tavor et al., 2016). However, using more thorough metrics and analysis, we demonstrate that published models yield predictions that are no better than group-average references. Furthermore, in this work, we revisit and offer a new step along this direction by proposing a methodological advancement, building on the modern tools of deep learning.

While recent advances in machine learning have enabled dramatic progress in a wide range of fields (LeCun et al., 2015), the functional MRI community has been more reluctant to adopt and promote deep learning (Bzdok and Yeo, 2017). Much of the hesitation in neuroimaging research can be attributed to lack of large-scale high-quality datasets. For example, in computer vision, datasets such as ImageNet Russakovsky et al. (2015) with millions of samples have made training high-capacity neural networks possible. Neuroimaging datasets, today, typically consist of hundreds of subjects or fewer. Furthermore, fMRI data can be highly noisy, due, in part, to motion or physiological artifacts (Power et al., 2015). The relatively low sample size and low SNR regime of neuroimaging makes training high-capacity neural networks challenging. Thus, we believe that it is important to implement neural network architectures that take full advantage of our knowledge about the data and neuroanatomy, while maximizing the available SNR.

**Fig. 1.**
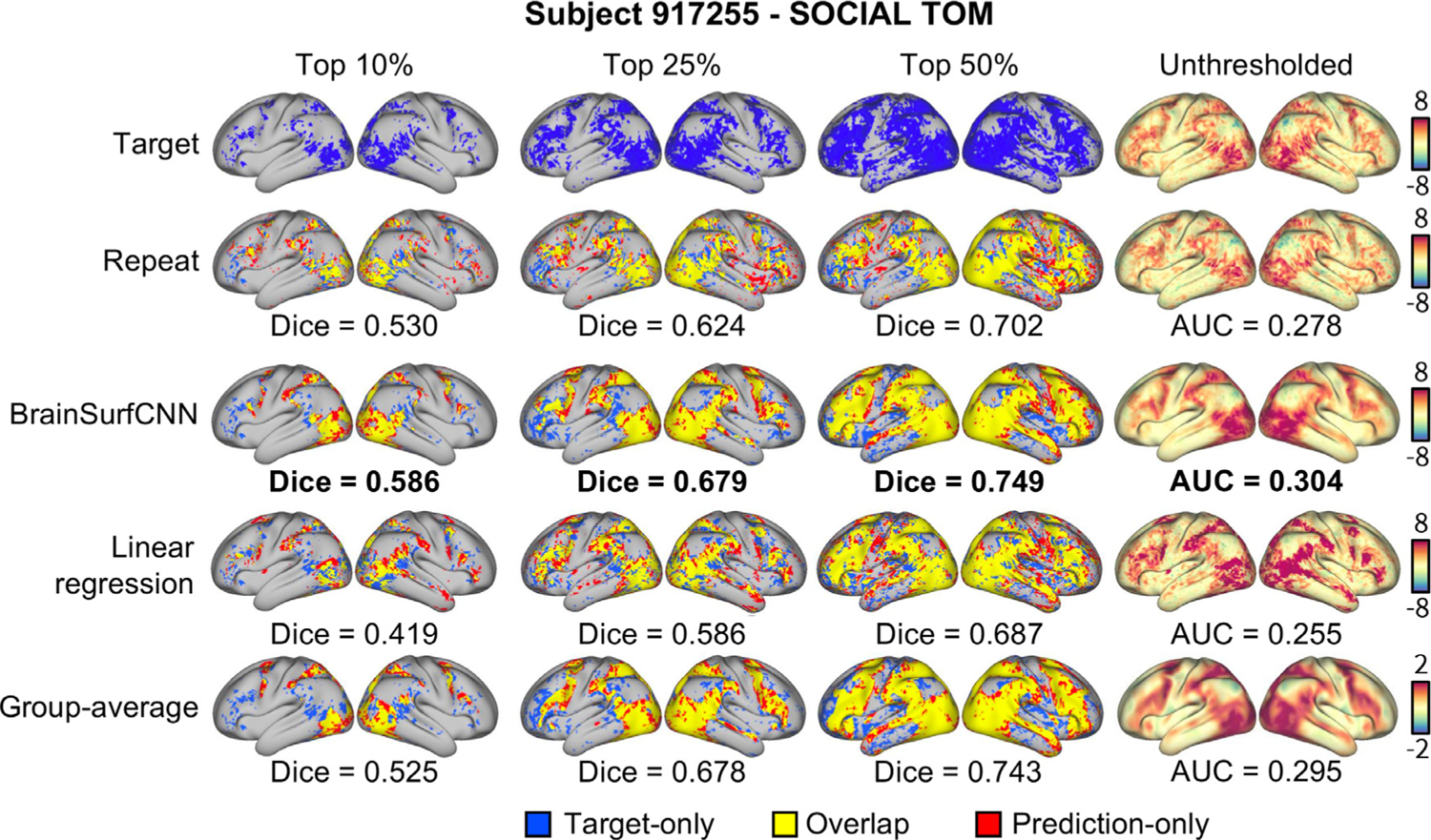
BrainSurfCNN model accurately predicts both coarse and fine-grained features of an individual subject’s task contrast in the HCP dataset. The leftmost three columns show the extent of overlaps between the target thresholded (fMRI-derived) activation map and predicted (from BrainSurfCNN and Linear regression) or reference maps (derived from repeat scan of the same individual performing the same task and group average task contrast). Blue represents the target activation, red represents the prediction or reference, and yellow is the overlap. The rightmost column shows the unthresholded activation maps, Dice overlap is indicated below the corresponding panel. “SOCIAL TOM” is short for “Social Cognition: Theory of Mind.

In this work, we propose a surface-based neural network, which we call BrainSurfCNN, to tackle the problem of predicting subject-specific task contrasts from resting-state functional connectivity. Most neural networks applied to brain imaging operate either in 3D volume, 2D slices, or with region-level vectorized data (e.g. Kamnitsas et al., 2017; Li et al., 2018). In the specific context of functional connectivity, an approach based on quantifying the similarity between regional fMRI time series, common approaches include working with 2D resting-state functional connectivity (rsFC) matrices (Kawahara et al., 2017), population-based graphs (Parisot et al., 2017), or multi-channel 3D volumes (Khosla et al., 2019a), which are treated as input to neural networks. Unlike rsFC matrices and population-level graphs, which make use of low-dimensional representations (pairwise functional connectivity between regions of interest, or ROIs), we use a much richer representation of functional connectivity (vertex-to-ROI). While our method of constructing functional connectivity is closely related to the multi-channel voxel-to-ROI 3D volumes in Khosla et al. (2019a), we work with a surface representation that captures the cortical geometry and allows modeling of fMRI signals on the gray-matter cortical sheet. Furthermore, inter-subject alignment and spatial smoothing on the cortical surface have been shown to better preserve the signal and yield more statistical power for detecting functional activations (Anticevic et al., 2008; Frost and Goebel, 2012; Klein et al., 2010). The fMRI field is increasingly recognizing the benefits of surface-based analysis and there has been a substantial shift from volume-based neuroimaging analysis to surface-based ones (Coalson et al., 2018), spearheaded by large-scale projects such as the Human Connectome Project (HCP) (Glasser et al., 2013). Building on these developments, in this paper we show that the proposed BrainSurfCNN achieves state-of-the-art predictions of individual task contrasts from resting-state functional connectivity, which is on par with the repeat reliability of the measured signal. Fig. 1 shows a representative example of the HCP’s “Social Cognition: Theory of Mind” task contrast for one subject. The first two rows are tfMRI derived maps (target, i.e., firs scan and repeat scan), followed by our BrainSurfCNN model’s prediction of the task contrast based on the resting functional connectome, presented in the third row. Dice scores, commonly used for evaluating accuracy of image segmentation, quantify the overlap with the target contrast map at different thresholds of activation. By measuring accuracy of prediction at different levels of activation, we can quantify the quality of the task contrast prediction at individual level, which must (1) resemble the smooth group-average pattern shared across subjects and (2) retain subject-specific detail that is reproducible across scans. We observe that our model’s predictions are remarkably consistent with the tfMRI measurements.

In our analyses, we further demonstrate that the BrainSurfCNN model generalizes well to novel tasks and new subjects using a transfer learning paradigm (Yosinski et al., 2014); we hypothesize this was possible due to the multi-task learning set-up used for training. Our results show that with the help of transfer and multi-task learning, neural networks applied to neuroimaging can be adapted beyond the original training dataset and generalize well to other contexts in which data might be limited.

## 2. Methods

### 2.1. Multi-channel vertex-to-ROI functional connectome

**Fig. 2.**
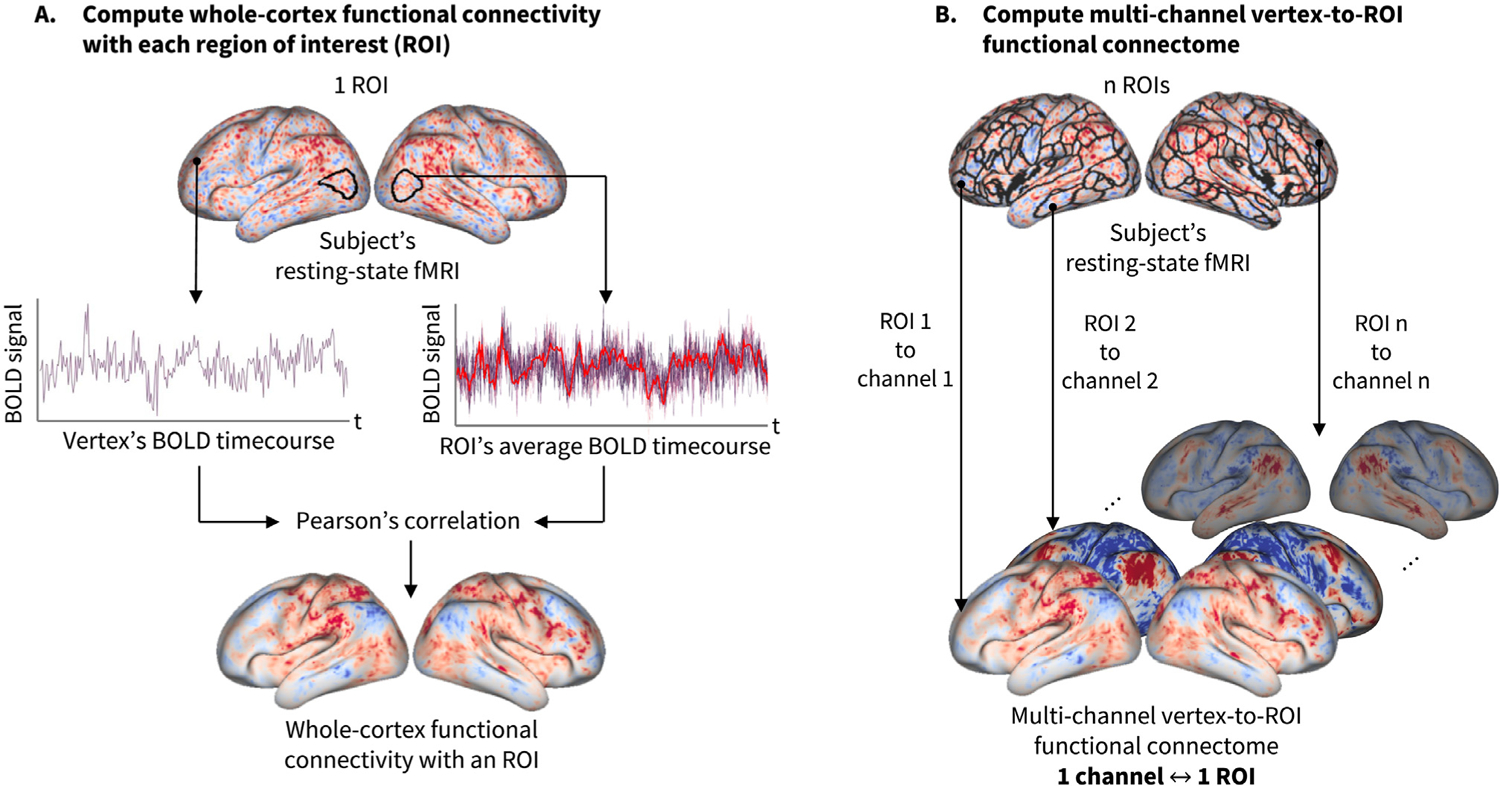
Multi-channel vertex-to-ROI functional connectomes computed from rsfMRI. The functional connectivity between a vertex and an ROI is computed as the Pearson’s correlation coefficient between a vertex’s rsfMRI timeseries and the average timeseries of an ROI. In our experiments, the functional connectomes were attached to the fs_LR 32k surface templates (Van Essen et al., 2012) with 84 channels, in which each channel corresponds to an ROI derived from independent component analysis (ICA) performed on the rsfMRI data of the training subjects.

Inputs to the predictive models are the functional connectomes, represented as multi-channel data attached to the icosahedral mesh vertices. Fig. 2 shows the construction of the multi-channel vertex-to-ROI functional connectomes used in our experiments. The vertex-to-ROI functional connectivity is given by:

(1) $$r_{ij}=\text{corr}\left(t_{i},{\bar{t}}_{j}\right),$$

where *r*_*ij*_ is the functional connectivity between the *i*-th vertex on the surface template and the *j*-th ROI, defined by the Pearson’s correlation coefficient *corr* (.) between the *i*-th vertex’s rsfMRI timeseries **t**_*i*_ and the average timeseries ${\bar{t}}_{j}$ of the *j*-th ROI. In our experiments, the surface template is the fs_LR 32k surface templates (Van Essen et al., 2012) with 32,492 vertices. The ROIs are parcels derived from group-level independent component analysis (ICA) (Smith et al., 2013) with 50 components computed from the resting-state fMRI timeseries of the training subjects (the test subjects were therefore excluded from the ICA). Each vertex is assigned to the parcel’s label corresponding to the component with the highest z-score value at the given location. Among the ROIs, 42 lie on the cortical surface. Each subject’s connectomes from the two hemispheres can be concatenated, resulting in a single input icosahedral mesh with the number of channels equalling twice the number of ROIs. Hence, the resulting functional connectome of each subject is a mesh of 32,492 vertices with 84 channel. Using the correlation of rsfMRI timeseries between vertices and ROIs to define brain functional connectivity is wide-spread practice in neuroimaging analysis, such as in seed-based maps (Cole et al., 2010; Greicius et al., 2003) or predictive modelling (Tavor et al., 2016). Compared to ROI-to-ROI functional connectomes (Cole et al., 2010) and dense vertex-to-vertex functional connectomes (Van Essen et al., 2013), vertex-to-ROI functional connectomes strike a balance between providing a high spatial resolution representation needed by predictive models (the focus of this work) while remaining computationally feasible. Note that alternative strategies for defining the ROIs can also be used, such as parcels from an atlas like the Schaefer et al. (2018) or Desikan et al. (2006) atlases. While the choice of ROIs would undoubtedly affect the model’s performance, we opted to use the same strategy as chosen by Tavor et al. (2016) for ease of comparison. Moreover, the surface-based representation of rsfMRI functional connectivity was chosen as it suits our research goal, i.e., localizing brain activation pattern on the cortical surface. We note that there might be more optimal representations for our application or other goals will benefit from different representations. For instance, localization of brain activity in subcortical regions will likely demand a volumetric representation (such as voxel-to-ROI representation in Khosla et al., 2019a). We leave the exploration of the choice of representation on predictive performance for future work.

### 2.2. BrainSurfCNN

**Fig. 3.**
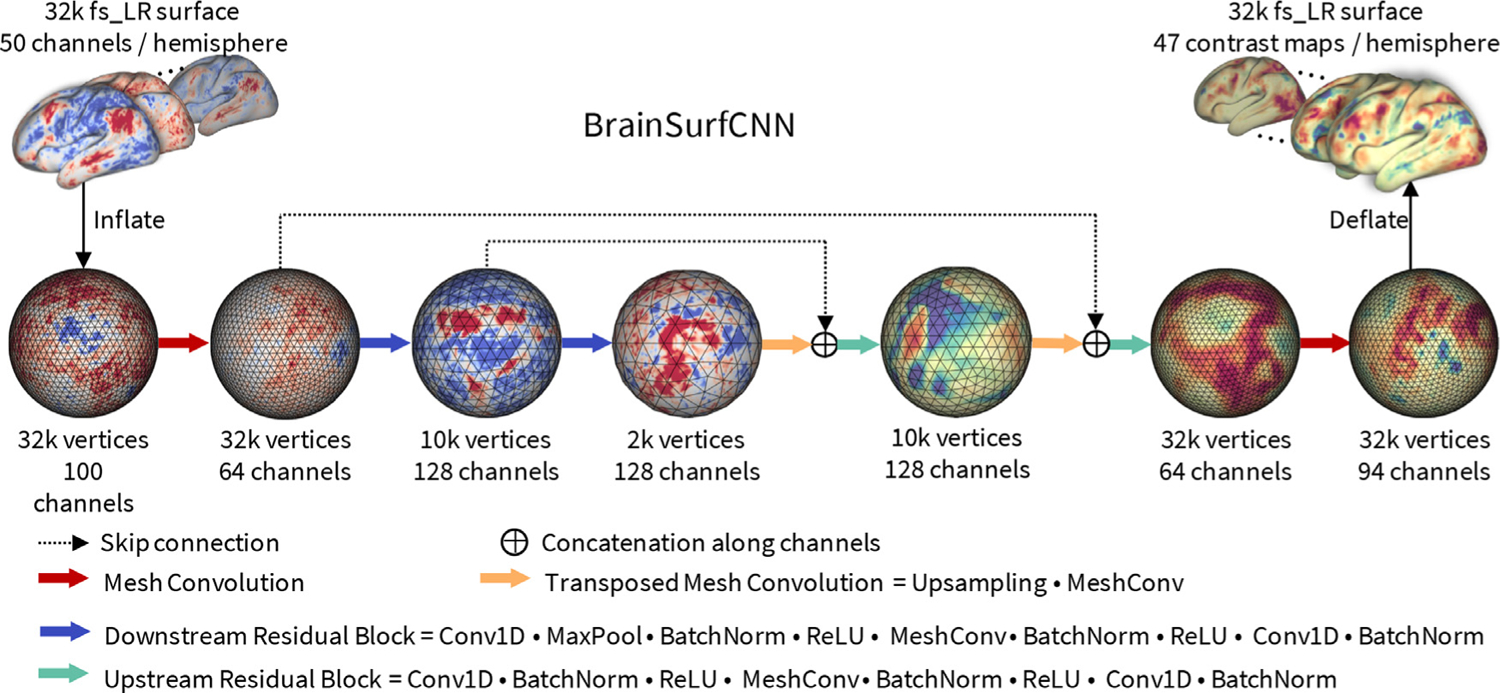
BrainSurfCNN model. BrainSurfCNN is a surface-based fully-convolutional neural network based on the U-Net architecture (Ronneberger et al., 2015) with spherical convolutional kernels (Chiyu et al., 2019). BrainSurfCNN’s input and output are multi-channel icosahedral fs_LR meshes (Van Essen et al., 2012). Each input channel is a functional connectivity feature, measured by Pearson’s correlation between the vertices’ timeseries and the average timeseries of an ROI. Each output channel corresponds to a fMRI task contrast.

Fig. 3 shows the proposed BrainSurfCNN model for predicting task contrasts from resting-state functional connectomes. BrainSurfCNN is based on the U-Net architecture (Milletari et al., 2016; Ronneberger et al., 2015) and uses the spherical convolutional kernel (Chiyu et al., 2019) to operate on the spherical mesh. The most notable feature in the U-Net architecture is the skip connections that copy features from the encoding arm (outputs from the downstream blocks in Fig. 3) to the inputs of the decoding arm (inputs for the upstream blocks in Fig. 3). The skip connections were found to improve U-Net predictive accuracy of fine-grained details in U-Net’s original task of image segmentation (Ronneberger et al., 2015). Our ablation study (Supplemental Table 1) shows skip connections similarly improves predictive quality in our image generation task. The input and output surfaces are fs_LR templates (Van Essen et al., 2012) with 32,492 vertices (fs_LR 32k surface) per brain hemisphere. The left and right hemispheres are symmetric in the fs_LR atlases, i.e., the same vertex index in both hemispheres corresponds to contralateral homologs. Thus, each subject’s connectomes from the two hemispheres can be concatenated, resulting in a single input icosahedral mesh with the number of channels equalling twice the number of ROIs. BrainSurfCNN’s output is also a multi-channel icosahedral mesh, in which each channel corresponds to one fMRI task contrast. This multi-task prediction setting promotes weight sharing across contrast predictions.

### 2.3. Reconstructive-contrastive loss

In training BrainSurfCNN, we minimize a reconstructive-contrastive (R-C) loss, which we describe here. The R-C loss is related to metric learning (Koch et al., 2015; Schroff et al., 2015), but adopted to the image generation task in the neuroscience domain. Given a mini batch of *N* samples $B=\left\{x_{i}\right\}$, in which $X_{i}$ is the target multi-channel contrast image of subject *i*, let ${\hat{x}}_{i}$ denote the corresponding prediction. The reconstructive-contrastive loss ${ℒ}_{RC}$ is defined as:

(2) $${ℒ}_{R}=\frac{1}{N}\overset{N}{\underset{i=1}{\sum }}d\left({\hat{x}}_{i},x_{i}\right)\;;\;{ℒ}_{C}=\frac{1}{\left(N^{2}-N\right)/2}\sum_{\underset{j\neq i}{x_{j}\in B_{i}}}d\left({\hat{x}}_{i},x_{j}\right)$$

(3) $${ℒ}_{RC}={\left[{ℒ}_{R}-\alpha \right]}_{+}+{\left[{ℒ}_{R}-{ℒ}_{C}+\gamma \right]}_{+}$$

where *d*(.) is a loss function (e.g. *l*^2^-norm). ${ℒ}_{R}$, *α* are the same-subject (reconstructive) loss and margin, respectively. ${ℒ}_{C}$, *γ* are the across-subject (contrastive) loss and margin, respectively. The combined objective ${ℒ}_{RC}$ encourages the same-subject error ${ℒ}_{R}$ to be within *α* margin, while pushing the across-subject difference ${ℒ}_{C}$ to be large such that $\left({ℒ}_{C}-{ℒ}_{R}\right)>\gamma$. How *α* and *γ* were set in practice is described below.

### 2.4. Baseline

#### 2.4.1. Linear regression

The linear regression baseline was implemented according to Tavor et al. (2016), as given by:

(4) $$y_{i}^{k}=X_{i}^{k}\beta_{i}^{k},$$

where $y_{i}^{k}$, $X_{i}^{k}$, $\beta_{i}^{k}$ are the vectorized activation pattern, input features, and regressor of the *k*th parcel in the *i* th subject, respectively. The 50-component parcellation derived from ICA, provided by HCP was used to compute the linear regression model. $y_{i}^{k}$ is a vector of length *n*_*k*_ - the number of vertices in the *k* ‘th parcel in both hemispheres. $X_{i}^{k}$ is a $n_{k}\times M$ functional connectivity matrix, where each element was computed as the Pearson’s correlation between a vertex and the average timeseries of each of the *M* ROIs (same timeseries used to compute BrainSurfCNN’s input). Following Tavor et al. (2016), a linear model was fit for every parcel and every task contrast of each training subject. Thus, after training of a given task contrast, for each parcel, there is a separate linear model fitted to every training subject. In order to compute predictions in a given test subjects, the weights of the fitted linear models were averaged across all training subjects to yield a single predictive model per parcel. We note that this corresponds to ensembling the linear models from all training subjects. The predicted activation pattern ${\hat{y}}_{\text{test }}^{k}$ of the *k*th parcel in an unseen test subject is computed as:

(5) $${\hat{y}}_{\text{test}}^{k}=X_{\text{test}}^{k}{\bar{\beta }}^{k}=X_{\text{test}}^{k}\frac{1}{N_{\text{train }}}\overset{N_{\text{train }}}{\underset{i=1}{\sum }}\beta_{i}^{k}=\frac{1}{N_{\text{train }}}\overset{N_{\text{train }}}{\underset{i=1}{\sum }}X_{\text{test}}^{k}\beta_{i}^{k},$$

where ${\bar{\beta }}^{k}$ is the averaged weights of the linear regression models for the *k*th parcel computed across *N*_train_ training subjects.

#### 2.4.2. Group-average contrasts

Different degrees of inter-subject variability manifest in different task contrasts. Such variability in prediction was a subject of interest for our study. Therefore, we used the group averages as a naive reference. The group-average task contrasts’ correspondence with individual contrasts would be low/high for tasks with high/low inter-subject variance. The group-average reference also epitomizes the most salient features of the activation map specific to a task condition, which any predictive model should capture.

#### 2.4.3. Repeat contrasts

We used repeat tfMRI scans (when available) to quantify the reliability of the target contrast maps and evaluate the predictive performance of BrainSurfCNN and the baselines. The repeat contrasts were compared to the first contrasts both in terms of overall correspondence (measured with Dice) and in the subject identification task.

## 3. Experiments

### 3.1. Human connectome project (HCP)

For our benchmarking experiments with a large dataset, we used the minimally pre-processed, FIX-cleaned 3-Tesla resting-state fMRI (rsfMRI) and task fMRI (tfMRI) of 1200 subjects from the Human Connectome Project (HCP). The dataset’s acquisition and preprocessing were described in Glasser et al. (2013), Smith et al. (2013) and Barch et al. (2013). rsfMRI data was acquired in four 15-min runs, each with 1200 time-points per subject. Group-level parcellations derived from spatial ICA were also released by HCP. We used ROIs from the 50-component parcellation for computing the functional connectomes. HCP’s tfMRI data comprises of 86 contrasts from 7 task domains (Barch et al., 2013), namely: WM (working memory), GAMBLING, MOTOR, LANGUAGE, SOCIAL RELATIONAL, and EMOTION. Following Tavor et al. (2016), redundant negative contrasts were excluded, resulting in 47 unique contrasts. Out of 1200 HCP subjects, 46 subjects also have repeat (second visit) 3T fMRI data. Including only subjects with all 4 rsfMRI runs and 47 tfMRI contrasts, our dataset comprised of 919 subjects for training/validation, and 39 independent subjects (with repeat scans) for evaluation.

### 3.2. Amsterdam open MRI collection (AOMIC)

AOMIC is a collection of multimodal brain imaging datasets from a relatively large number of subjects (Snoek et al., 2021). For our experiments with transfer learning, we used two AOMIC datasets: PIOP1 and PIOP2 (PIOP stands for Population Imaging of Psychology). PIOP1 consists of 6 min of rsfMRI (480 timepoints at 0.75-second TR) and tfMRI measured from five tasks, namely “Emotion matching”, “Gender Stroop”, “Working memory”, “Face perception”, and “Anticipation”, for 216 subjects. PIOP2 consists of 8 min of rsFMRI (240 timepoints at 2-second TR) and tfMRI collected from three tasks, namely “Emotion matching”, “Working memory” and “Stop signal”, for 226 subjects. AOMIC data are organized according to the Brain Imaging Data Structure (BIDS) (Gorgolewski et al., 2016). RsfMRI data from AOMIC were preprocessed in the volumetric space using the default pipeline from fmriprep Esteban et al. (2019), a Nipype Gorgolewski et al. (2011) based tool focused on transparent and reproducible preprocessing of neuroimaging data. The autogenerated details of the preprocessing steps were reproduced in the Supplemental Methods. Preprocessed volumetric rsfMRI and tfMRI data were projected to the fs_LR surface via fsaverage template (Wu et al., 2018).

### 3.3. Individual brain charting (IBC)

To demonstrate the flexible domain adaptation our multi-task learning framework affords, we used data from the Individual Brain Charting (IBC) project (Pinho et al., 2020). IBC dataset includes fMRI data from 12 subjects and 180 task contrasts, 43 of which are also studied in the HCP. IBC data was preprocessed in the volume using fmriprep and projected to the fs_LR surface using the same procedures applied to the AOMIC datasets.

### 3.4. Experimental setup

#### 3.4.1. Data augmentation and test-time ensembling

In the HCP dataset, each subject has 4 rsfMRI runs with 1200 time-points each. As stable functional connectomes can be estimated from fewer than 1200 time-points (Finn et al., 2015), we computed a functional connectome from each contiguous half (600 time-points) of every run, resulting in 8 input samples per subject. During BrainSurfCNN training, one connectome was randomly sampled for each subject. Thus, the model was presented with 8 slightly different samples per subject. At test time, 8 predictions were computed for each subject and then averaged for a final prediction. For the AOMIC dataset, 4 functional connectomes were computed for each subject from 4 contiguous segments of 120 timepoints. For the IBC dataset, 2 connectomes were computed for each subject, each connectome was estimated from a randomly sampled contiguous segments of 600 timepoints.

#### 3.4.2. Training schedule

On the HCP dataset, BrainSurfCNN was first trained for 50 epochs with a batch size of 2 with mean squared error (MSE), i.e. reconstructive loss *L*_*R*_ in Eq. (3), using Adam optimizer. With this setup, training took about 5 h on one NvidiaRTX GPU (in comparison, training the linear regression baseline took 2 h when run in parallel on 20 AMD EPYC 7642 48-Core processors). Upon convergence, the average reconstructive loss *L*_*R*_ and constrastive loss *L*_*C*_ were computed on all training subjects. These values, were then used as initial values for the margins *α* and *γ* in Eq. (3). The initialization procedure forces the model to not deviate from the existing reconstructive error while optimizing for the contrastive loss. Training then continued for another 50 epochs, with the same-subject margin *α* halved and across-subject margin *γ* doubled every 20 epochs, which encourages the model to refine further over time. For transfer learning experiments with the AOMIC and IBC datasets, the best checkpoint of the model when training on HCP dataset with MSE was used as the initialization for finetuning. The hyperparameter values were set to achieve the lowest MSE on the validation samples of each dataset. This approach of initializing with pretrained weights and hyperparameter adjustment is widely used to handle dataset shift (Goodfellow et al., 2016). The finetuning was conducted with MSE as the objective function (i.e., with reconstructive loss only), as we did not see any benefit of including the constrastive loss in smaller datasets.

#### 3.4.3. Transfer learning

We investigated BrainSurfCNN’s generalizability beyond the HCP dataset on which the model is originally trained using two independent datasets, namely the AOMIC and IBC (Sections 3.2 and 3.3). Both datasets are significantly smaller in size and represent some degree of dataset shift from the HCP dataset. Due to their different natures, two different transfer learning strategies were explored. For the AOMIC datasets, the target contrasts were largely non-overlapping with the HCP contrasts. So, to handle this situation, we implemented a new output layer for BrainSurfCNN, which was randomly initialized. This output layer has the same number of output channels as the number of target contrasts in AOMIC. Weights of remaining (backbone) layers, on the other hand, were initialized with the HCP trained values. All the layers’ weights were then finetuned with the new training samples. For the IBC dataset, while there are significantly smaller number of training samples, there is some overlap between the IBC tasks and the HCP tasks. We wanted to exploit this overlap, to be able to predict all HCP contrasts in the IBC dataset, while adapting to the dataset shift. Hence, the output (deconvolutional) layer of the pretrained BrainSurfCNN was frozen and reused on the task contrasts shared by the HCP and IBC dataset, while the weights in the model backbone were finetuned using the loss function computed for the channels that correspond to the overlapping task contrasts in computing (and backpropagating from).

#### 3.4.4. Finetuning on medium-sized AOMIC datasets

We explored model predictions on two Population Imaging of Psychology (PIOP) datasets under the Amsterdam Open MRI Collection (AOMIC) (Snoek et al., 2021). Each dataset has both resting-state and task-based fMRI data of healthy participants; PIOP1 has 216 subjects with 5 tasks each and PIOP2 has 226 subjects with 3 tasks each. All AMOIC data, including raw and derivatives, are publicly available (Snoek et al., 2021).

There are significant differences between the HCP and AMOIC PIOP datasets, including differences in the task paradigms and scanning procedures. In addition, while the resting-state and task-based fMRI in the HCP dataset was preprocessed directly on the fs_LR surface space (Van Essen et al., 2013), the resting-state and task-based fMRI in the PIOP datasets were preprocessed in the volumetric MNI space with fmriprep (Esteban et al., 2019). As an extra preprocessing step, resting-sate and task-based fMRI data (t-stats maps) from the PIOP datasets were projected to the fs_LR surface templates via the fsaverage space for all subsequent training and prediction (Wu et al., 2018)(details in the Methods section). For both the PIOP1 and PIOP2 datasets, 50 subjects were held out for testing, leaving the rest for training and validation. The partitions were selected to ensure that all training and validation subjects have both resting-state and all task contrasts, but not all test subjects necessarily have all task contrasts.

Two versions of BrainSurfCNN were assessed, one was trained denovo on the training subset of each PIOP dataset using a random intialization - BrainSurfCNN (random init) - while another was finetuned from the HCP-pretrained model using the same PIOP data (BrainSurfCNN finetuned). The group-average task contrasts and linear model predictions were used for comparison.

#### 3.4.5. Finetuning on HCP task contrasts in IBC dataset

We want to investigate the representation shared across predicted task contrasts in the multi-task learning setup of BrainSurfCNN. As all predicted outputs of the multi-contrast BrainSurfCNN model share the same backbone network, which excludes the last deconvolutional layer (Fig. 3), we hypothesize that one can achieve domain adaptation via fine-tuning the backbone model based on shared (common) target contrasts. This will yield a model that can produce predictions for the tasks that are available during pre-training (e.g., HCP) but unavailable in the new domain dataset.

We explore this hypothesis by finetuning the backbone of the HCP-pretrained BrainSurfCNN model using a new dataset, the Individual Brain Charting (IBC) (Pinho et al., 2020; 2018). At the time of our analysis the IBC dataset has 12 subjects with both resting-state and task contrast fMRI scans. As the IBC project aims to densely sample cognitive processes, the dataset covers a wide range of task paradigms per subject, including some that are similar but not identical to HCP tasks (see Supplemental Table 2 for task contrasts that are similar between HCP and IBC datasets). For example, IBC “Language” task stimuli are the French translation of the English stimuli used in the original HCP tasks. Together with differences in scanning and preprocessing protocols, there are significant domain shifts between the HCP and IBC datasets even for the same task paradigms. The subjects perform each task in two sessions, one with anterior-posterior (AP) and the other with posterior-anterior (PA) phase encoding during acquisition (Pinho et al., 2018). In our experiments, the task contrasts derived from the AP sequence is the measured target, while the contrasts from the PA sequence is treated as the reliability reference (analogous to the repeat contrasts in the HCP dataset). We adapt the BrainSurfCNN model pretrained on the HCP data to the IBC dataset by only finetuning the backbone of the model in a leave-one-task-out procedure. Here the predicted IBC task (which itself consists of multiple contrasts and has a corresponding HCP task) is treated as unseen and the remaining HCP contrasts are used as the shared target contrasts for finetuning the BrainSurfCNN backbone. The backbone-finetuned BrainSurfCNN is compared against the multi-contrast and single-contrast BrainSurfCNN, the linear regression models that were only trained on HCP dataset (no finetuning) and the group-average contrasts.

#### 3.4.6. Evaluation metrics for predicted images

Dice score Dice (1945) is used to measure the extent of overlap between a predicted contrast and the target contrast map for a given percentage of most activated vertices. At a given threshold of *x* %, Dice score is computed as:

(6) $$\text{Dice}(x)=\frac{2\left|\text{Prediction}(x)\cap \text{Target}(x)\right|}{\left|\text{Prediction}(x)\right|+\left|\text{Target}(x)\right|},$$

where |Prediction(x)| denotes the number of top *x*% most activated vertices in the predicted contrast map, |Target(x)| denotes the number of top *x*% most activated vertices in the contrast, and |Prediction(x)∩Target(x)| denotes the number of vertices that overlap between the predicted and target map at the given threshold. At a lower threshold (e.g., when looking at 5% most activated vertices), the Dice score measures the correspondence of the fine-grained details between the target and predicted contrasts. At higher thresholds (e.g. 50% most activated vertices), this metric quantifies the global agreement of the anatomical distributions. By integrating Dice scores over a range of thresholds (5% to 50% most activated vertices at 5% interval, using the composite trapezoidal rule), we produce a summary measure - area under the Dice curve (Dice AUC) - for the quality of a model prediction.

#### 3.4.7. Quantifying identification accuracy

Dice AUCs were computed between the models’ predicted individual task contrast maps and the tfMRI-derived target contrast maps of all subjects. This results in a 39 by 39 AUC matrix for each contrast, where each entry is the Dice AUC between a subject’s predicted contrast (column) and a target contrast map (row), of same or another subject. The difference between diagonal and average off-diagonal values (Dice AUC between a subject’s predicted contrast map with the target contrast map derived from another subject’s tfMRI) indicates how much better one subject’s prediction corresponds with the subject’s own tfMRI-derived contrast compared to other subject contrasts. In other words, the *i*-th subject is identifiable among all test subjects using the predicted contrast if the *i*-th element of the *i*-th row has the highest value. For a given task contrast and prediction model, we compute subject identification accuracy as the fraction of subjects with a maximum at the diagonal.

## 4. Results

### 4.1. BrainSurfCNN’s predictive accuracy approaches reliability

**Fig. 4.**
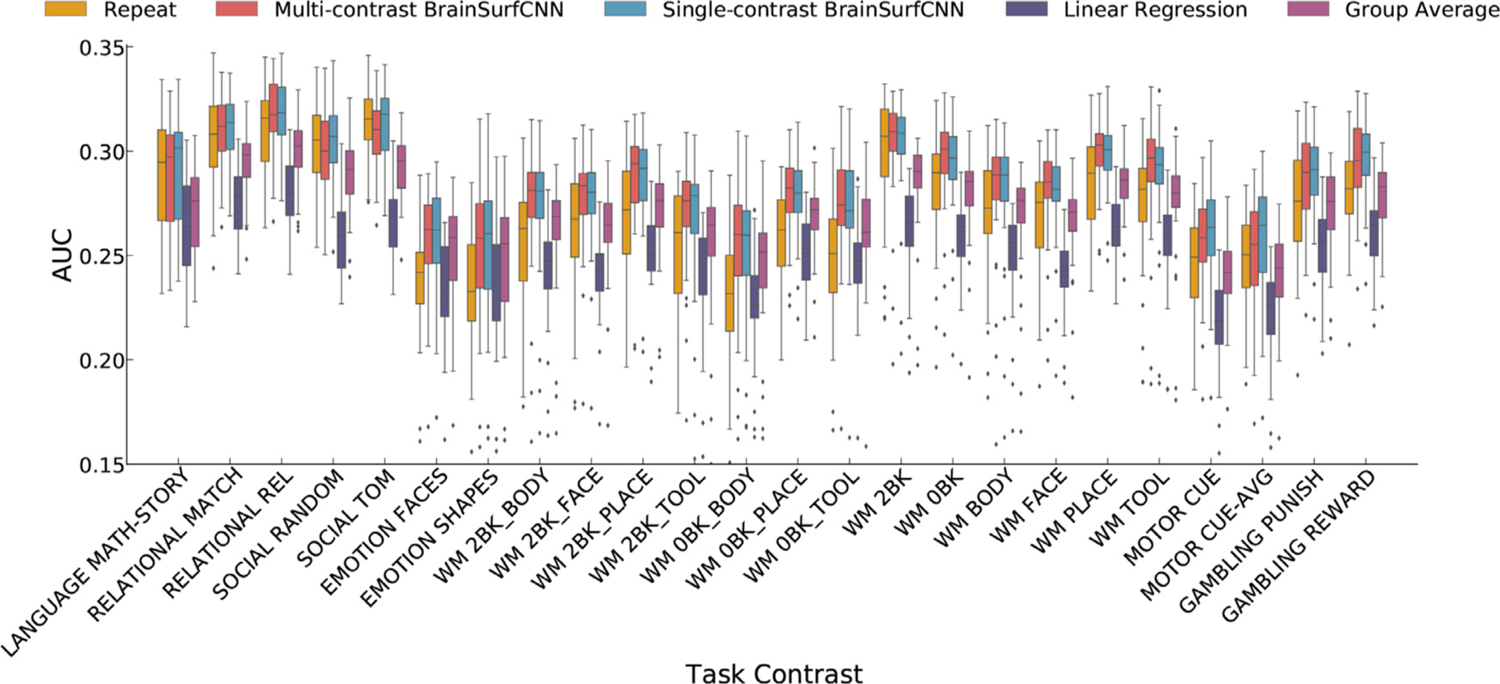
BrainSurfCNN prediction is better than the linear regression prediction and group-average contrast map while approaching the reliability of the task contrast (measured by the correspondence with the repeat task contrast map) across the most individual-level reliable HCP task contrasts (whose target-repeat reliability AUC is higher than the group-average). Quality of prediction is measured as the area under the curve (AUC) of Dice overlap between target and predicted or reference thresholded activation maps (Fig. 5). “REL”, “AVG” are short for “Relational” and “Average”, respectively. “WM” is short for “Working memory” task; “0BK” and “2BK” are short for “0-back” and “2-back” conditions of the working memory task, respectively. The list of all 47 HCP task contrasts are available in Supplemental Table 2.

We applied BrainSurfCNN to the Human Connectome Project (HCP) dataset to assess the model’s predictive performance. Nine hundred and nineteen subjects were used for training and validation, while the 39 HCP subjects with a repeat scan were used for testing. Two formulations of BrainSurfCNN were evaluated. The multi-contrast BrainSurfCNN was trained to make predictions for all 47 task contrasts simultaneously, while single-task BrainSurfCNN models were trained separately for each task contrast. We compared BrainSurfCNN predictions against two baselines; the group-average task contrasts and a linear regression model (Tavor et al., 2016). In addition, we used the repeat tfMRI scans to assess the reliability of each subject’s task contrast, which was quantified as the Dice AUC between the maps derived from the target and repeat scans. Supplemental Figs. 6 and 7 show the predictive performance of the different models using either the rsfMRI input from the first or repeat (second) scan as inputs, and the task contrasts from the repeat (second) scan and the average contrasts of both scans as targets. The same trend of predictive performance is observed in all three setups with both the Dice AUC and *R*^2^ metrics. We also observe that using either rsfMRI input from the first or repeat (second) scan yield similar predictive accuracy for both BrainSurfCNN and linear regression models. Using either tfMRI task contrasts from the first or repeat (second) scan as targets also yield similar predictive accuracy for both models. Furthermore, the average target task contrasts (average of the contrasts from the first and second scan) yields better predictive accuracy than first or repeat scan targets for both models. This suggests that the tfMRI contrasts contain scan-specific noise that can be reduced via averaging. Out of 47 HCP task contrasts, 24 had a target-repeat reliability AUC higher than the group-average and thus were considered individual-level reliable contrasts. The reported measure of reliability is based on an empirical measurement and independent of any predictive model. We did not focus on unreliable task contrasts because the low signal-to-noise ratios would make it challenging to train a prediction model on these targets. Without a reliable target, the evaluation of the predictions would also be challenging. Fig. 4 shows the Dice AUC of the different models across the 24 individual-level reliable HCP task contrasts. Across the 24 individual-level reliable task contrasts and all test subjects, multi-contrast BrainSurfCNN’s average AUC (0.2828) is on par with the single-contrast BrainSurfCNN models’ average AUC (0.2834) for all but 6 reliable contrasts), but statistically significantly higher than the group-average contrasts (0.2693) (*p* < 10^−4^ for all reliable contrasts), and the linear model’s baseline predictions (0.2500) (*p* < 10^−4^ for all reliable contrasts). BrainSurfCNN also outperforms the average AUC of repeat contrasts (0.2697) (*p* < 10^−4^ for 15 out of 24 individual-level reliable contrasts). All p-values are estimated from paired 2-tail *t*-test and reported in Supplemental Table 5 together with the average Dice AUC of each task contrast in Supplemental Table 4. The same trend of predictive accuracy can be observed when another metric, whole-brain R2 (Dohmatob et al., 2021), is used (Supplemental Fig. 2, which suggests the improved performance of BrainSurfCNN is not merely a consequence of the choice of Dice as our evaluation metric.

**Fig. 5.**
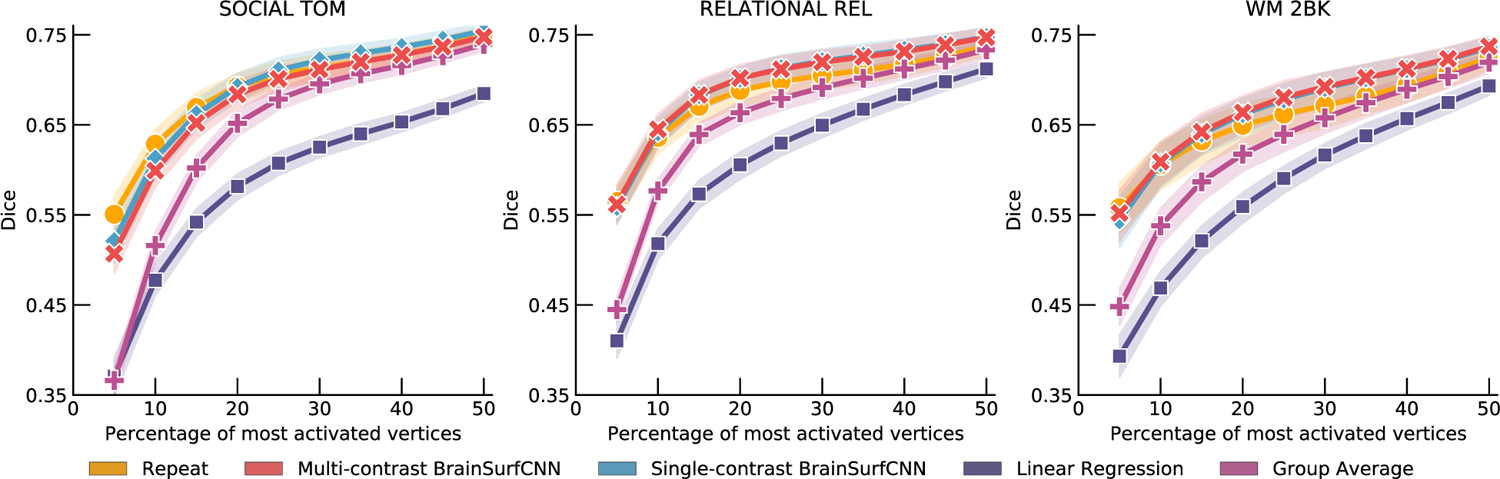
BrainSurfCNN’s predicted task contrasts are comparable to contrasts from repeat task contrasts. For the three most reliable HCP task contrasts (highest overlap with repeat scan), namely “SOCIAL TOM” (social cognition: theory of mind), “RELATIONAL REL” (relational processing), “WM 2BK” (working memory: 2-back), BrainSurfCNN’s Dice overlap with the true activation maps is close to the reliability limit.

The three contrasts (from different tasks) with the highest reliability Dice AUC are “SOCIAL TOM” (reliability AUC = 0.312), “RELATIONAL REL” (reliability AUC = 0.310), and “WM 2BK” (reliability AUC = 0.297). Fig. 5 shows the Dice score curves for these contrasts. The Dice scores of BrainSurfCNN closely approach the reliability Dice scores across all thresholds, suggesting that the BrainSurfCNN prediction well captures the individual-level details of task contrasts. Conversely, the agreement between the group-average and measured target task contrasts is lower than the repeat measurement, when only a small fraction of top activated vertices are considered, but approaches the reliability score when computed over the majority of vertices. This suggests that the group-average map indeed captures large-scale patterns, but smooths over the individual-level fine details. Supplemental Figs. 1 to 5 shows the Dice and AUC for all 47 HCP task contrasts.

**Fig. 6.**
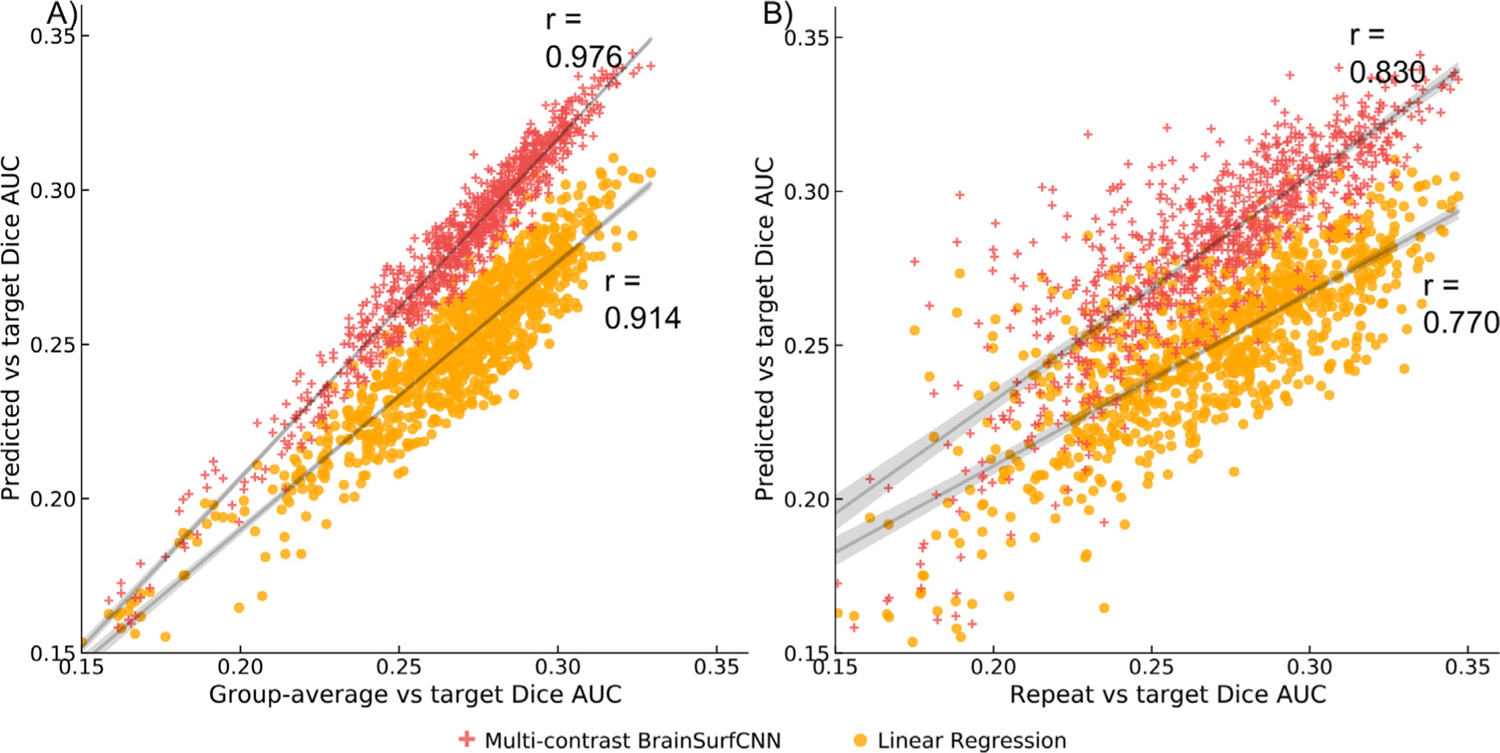
Relationship between models’ predictive quality and the correspondence of target and reference (repeat or group-average) task contrasts. In each plot, x-axis is the Dice AUC between the reference and the target contrast maps for all test subjects across all individual-level reliable HCP task contrasts. Y-axis is the Dice AUC between the predicted and target individual contrast maps. Each plot also shows the correlation coefficients between the predicted vs target AUCs and the reference vs target AUCs. Overall, the graphs indicate that both BrainSurfCNN and linear regression models make better predictions for individual task contrasts that overlap better with the group-average maps. Both models also have better predictive accuracy for individual task contrasts with higher intra-subject reliability. Such trends are more prominent for BrainSurfCNN than the linear regression baseline.

Fig. 6 shows the relationship between predictive accuracy and the correspondence of individual target contrasts with reference maps over all test subjects and reliable HCP task contrasts. As shown in Fig. 6 A, the Pearson’s correlation coefficient between target versus predicted Dice AUCs and target vs group-average Dice AUCs for the linear regression model is *r* = 0. 874, and *r* = 0. 976 for BrainSurfCNN. This indicates that both models better predict individual task contrasts that are more consistent with the group-average, but the effect is more pronounced for BrainSurfCNN. On the other hand, considering the correlation coefficient between target versus predicted Dice AUCs and target versus repeat Dice AUCs (Fig. 6 B), linear regression baseline yields *r* = 0. 737 while BrainSurfCNN yields *r* = 0. 845. This measure also indicates that the quality of prediction by BrainSurfCNN better correlates with the replicability of individual task contrasts than the linear regression model. We also compute the Pearson’s correlation coefficients for all task contrasts of each test subject and perform paired 2-tail *t*-test on the Fisher z-transformed correlation coefficients. For target versus predicted Dice AUCs and target vs group-average Dice AUCs, the average correlation coefficients per test subject are 0.97 and 0.92 for BrainSurfCNN and linear regression, respectively, with *p* = 6.7 × 10^−13^. For target versus predicted Dice AUCs and target vs repeat Dice AUCs, the average correlation coefficients per test subject are 0.85 and 0.77 for BrainSurfCNN and linear regression, respectively, with *p* = 8.9 × 10^−9^. The trends of both auxiliary correlation measures corroborate our claim that BrainSurfCNN’s superior predictive accuracy can be attributed to the model’s capability to learn both the gross topology of a task contrast, as well as infer the intricate, but replicable variation intrinsic to each individual. Furthermore, these results perhaps reiterate a rather unsurprising reminder that the performance of any predictive model is bounded by the noise ceiling inherently present in the data. Thus, references such as comparison with repeat measurements (if possible) or population averages can be a proxy for the noise level and can offer yardsticks for evaluating any predictive model.

### 4.2. BrainSurfCNN predictions are highly subject-specific

**Fig. 7.**
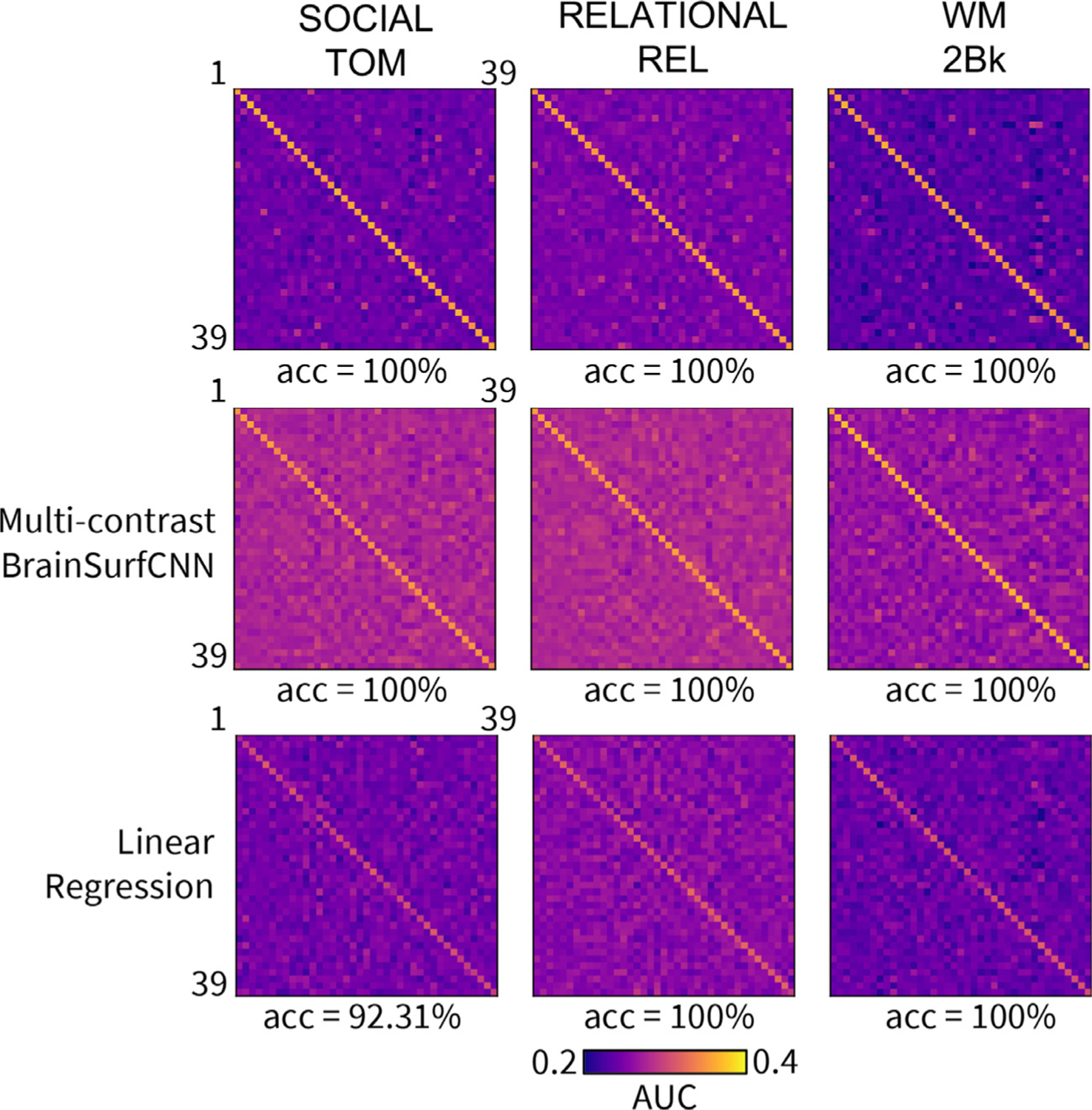
AUC values of prediction versus target measured subject contrasts for 3 most reliable HCP task contrasts, across 39 test subjects. Each row corresponds to a subject’s target task contrast and each column corresponds to the subject’s prediction. The accuracy score below each matrix is the identification accuracy of the model’s prediction for each task.

**Fig. 8.**
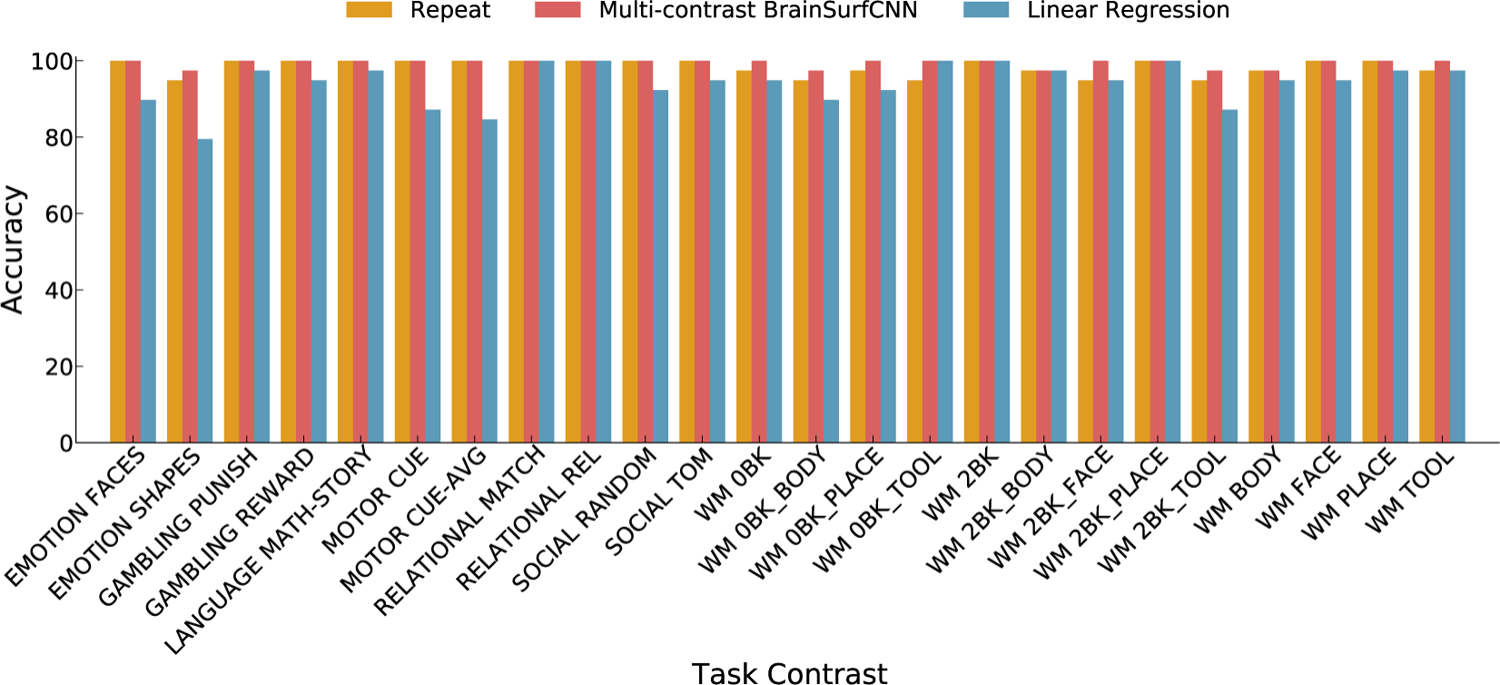
Subject identification accuracy of predictions for each of 24 individual-level reliable HCP task contrasts. A task contrast prediction accurately identifies a subject if it matches the subject’s target contrast better (in Dice AUC) than any other subject in the test set. This is equivalent to the diagonal element having the highest value in a column of a Dice AUC matrix such as Fig. 7. AUC matrices of all reliable HCP task contrasts are shown in Supplemental Fig. 8 to 10.

Fig. 7 displays the agreement (AUC) across individual subjects’ predicted or repeat measured contrasts (columns) and their measured target contrasts (rows). A subject’s prediction is considered identifiable if it achieves the highest AUC with the subject’s own target contrast, i.e. the diagonal element has the highest value in the column. We compute the identification accuracy as the fraction of subjects that are identifiable. Across the three most reliable task contrasts (SOCIAL TOM, RELATIONAL REL, and WM 2BK), both the repeat scan and BrainSurfCNN predictions yield a 100% identification accuracy, with the linear regression baseline yields a lower accuracy for “SOCIAL TOM” task contrast. Supplemental Figures. 8, 9 and 10 show the AUC matrices of prediction versus target subject contrasts of all 24 individual-level reliable HCP task contrasts for repeat contrasts, multi-contrast BrainSurfCNN and linear regression models, respectively. Fig. 8 shows the identification accuracy across 24 individual-level reliable HCP task contrasts for BrainSurfCNN, linear regression models, and the repeat reference. Task contrasts predicted by BrainSurfCNN consistently have better subject identification accuracy than the linear regression models (*p* = 3 × 10^−5^ with paired 2-tail *t*-test over the 24 contrasts), with 100% accuracy over all but the “EMOTION SHAPES”, “WM 0BK_BODY”, “WM 2BK_BODY”, “WM 2BK_TOOL” and “WM BODY” contrasts. It is interesting that BrainSurfCNN even outperforms the repeat reference in subject identification accuracy for 8 of the task contrasts (*p* = .0048 with 2-tail paired *t*-test). This is possibly due to the smoothness of predicted contrasts, which might favor BrainSurfCNN model in the multi-threshold Dice metrics (and consequently Dice AUC and identification accuracy). Similarly, measures of target-repeat overlap would likely improve with more aggressive smoothing that would remove more of the intra-subject noise. The same trend of predictive accuracy can be observed when another metrics, whole-brain *R*^2^ (Dohmatob et al., 2021) is used (Supplemental Figs. 2, 7, which suggests the improved performance of BrainSurfCNN is not merely a consequence of the choice of Dice as evaluation metrics.

**Fig. 9.**
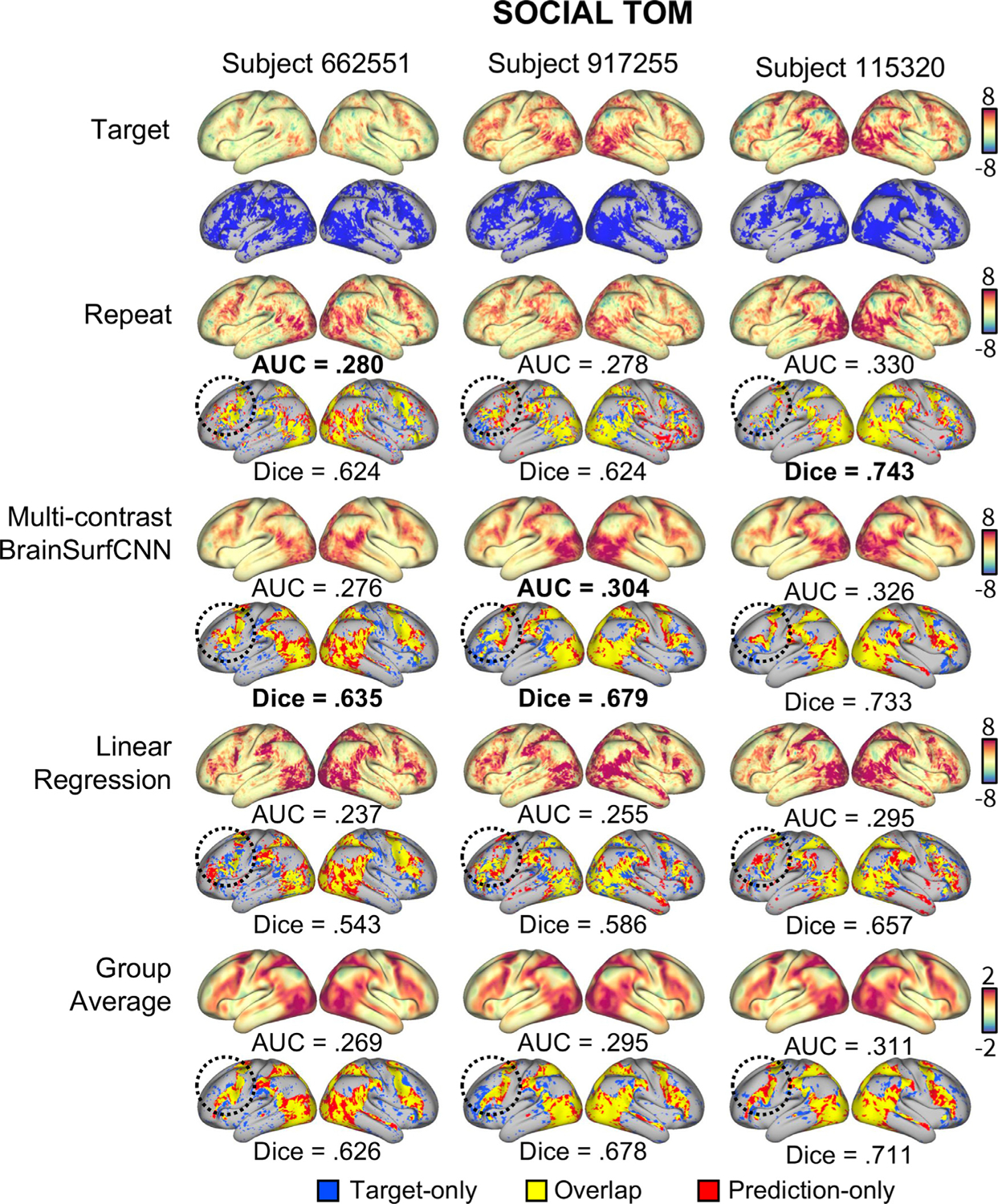
Measured and predicted “SOCIAL TOM” (Social cognition, theory of mind) task contrast for three representative subjects in the HCP dataset. Each row shows both the unthresholded activation maps (top) and thresholded maps of the top 25% most activated vertices (bottom). Blue indicates activation in the measured contrast, red is the predicted or reference activation and yellow is the overlap. The circled areas show activation patterns of the prefrontal cortex distinct to each subject that are replicable in both the repeat contrasts and BrainSurfCNN prediction.

Fig. 9 shows the measured (target) and predicted task contrasts for 3 subjects, including both the unthresholded contrast maps (top rows) and the top 25% most activated vertices (bottom rows). The task contrast in consideration is “SOCIAL TOM”, which has the highest reliability. The three subjects, “662551”, “917255”, and “115320” have the 10th, 50th, and 90th percentile “target vs. group-average” AUC among the test subjects, respectively. Thus, these three subjects represent varying degrees of deviation from the typical (group-average) contrast. Focusing on the prefrontal cortex, there are subject-specific activation patterns that appear in the repeat scans. For instance, the replicable activation pattern of the prefrontal cortex in subject “115320” is more laterally dominant, and subject “917255” has more activation in the inferior prefrontal cortex. On the other hand, the activation pattern of subject “662551” is more sparsely distributed in the prefrontal cortex. Such subject-specific characteristics are captured in the predictions computed by BrainSurfCNN, also reflected by a high Dice overlap between the predicted task contrast and the measured target contrasts for the top 25% most activated vertices. It is also worth noting that the linear regression baseline from Tavor et al. (2016), was not better than the group-average reference, both visually (Fig. 9) and quantitatively (Fig. 4). While the linear regression model indeed predicts variability in the task contrasts unique to each subject (evident by the decent identifiability shown in Fig. 8), the predicted maps might deviate too much from the prototypical topology of activation (estimated by the group-average), which can be observed in Fig. 9.

### 4.3. Transfer learning improves pretrained BrainSurfCNN’s predictive performance on smaller datasets

#### 4.3.1. Amsterdam OpenfMRI PIOP1

**Fig. 10.**
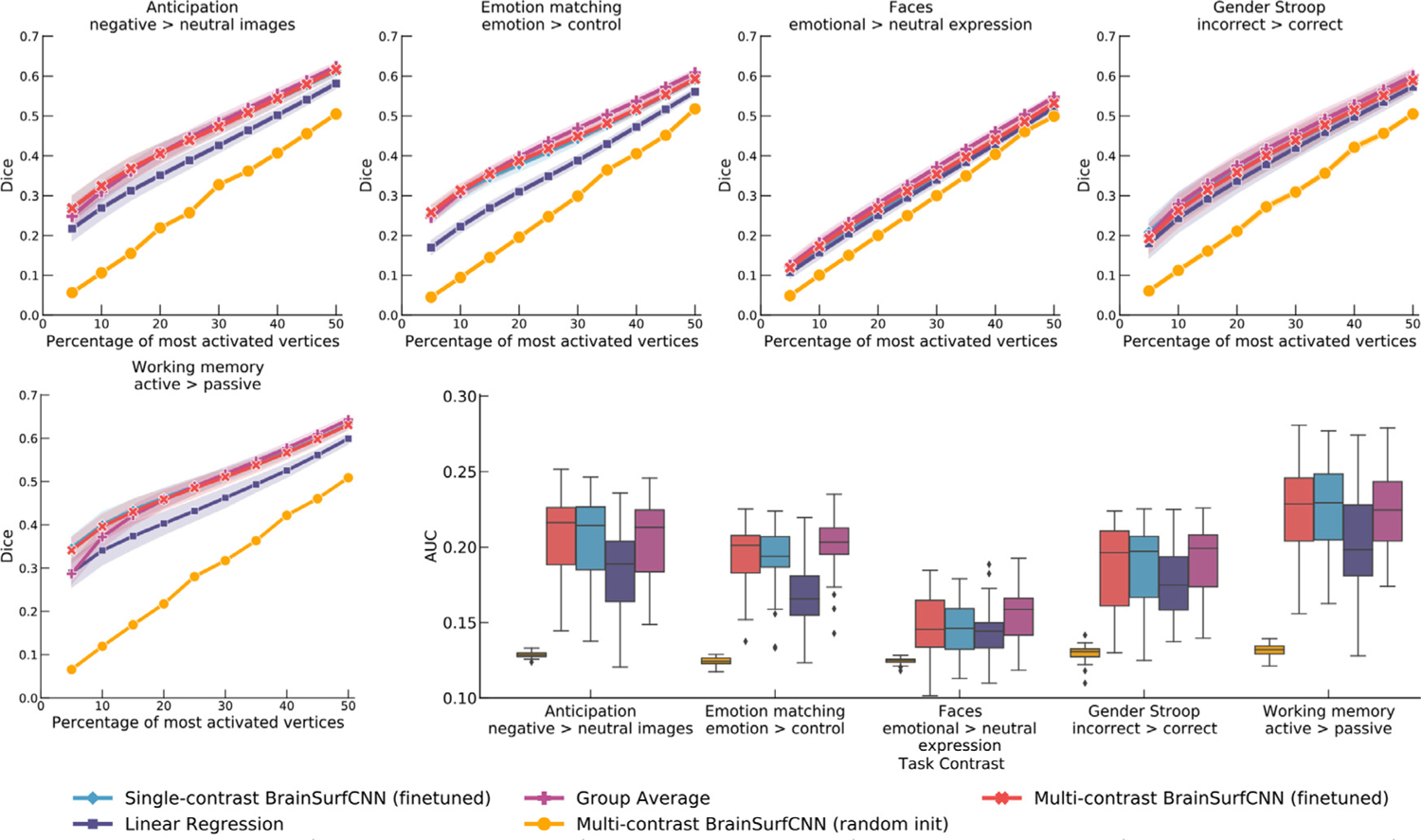
Quality of various model predictions (Dice and AUC) for the Amsterdam PIOP1 dataset. BrainSurfCNN cannot learn effectively if trained from random intialization, resulting in low Dice scores across all thresholds and low overall Dice AUC. However, by finetuning a pretrained model (on HCP data), BrainSurfCNN significantly improves its predictive accuracy and surpasses the linear regression baseline in both the single-contrast and multi-contrast learning setting.

**Fig. 11.**
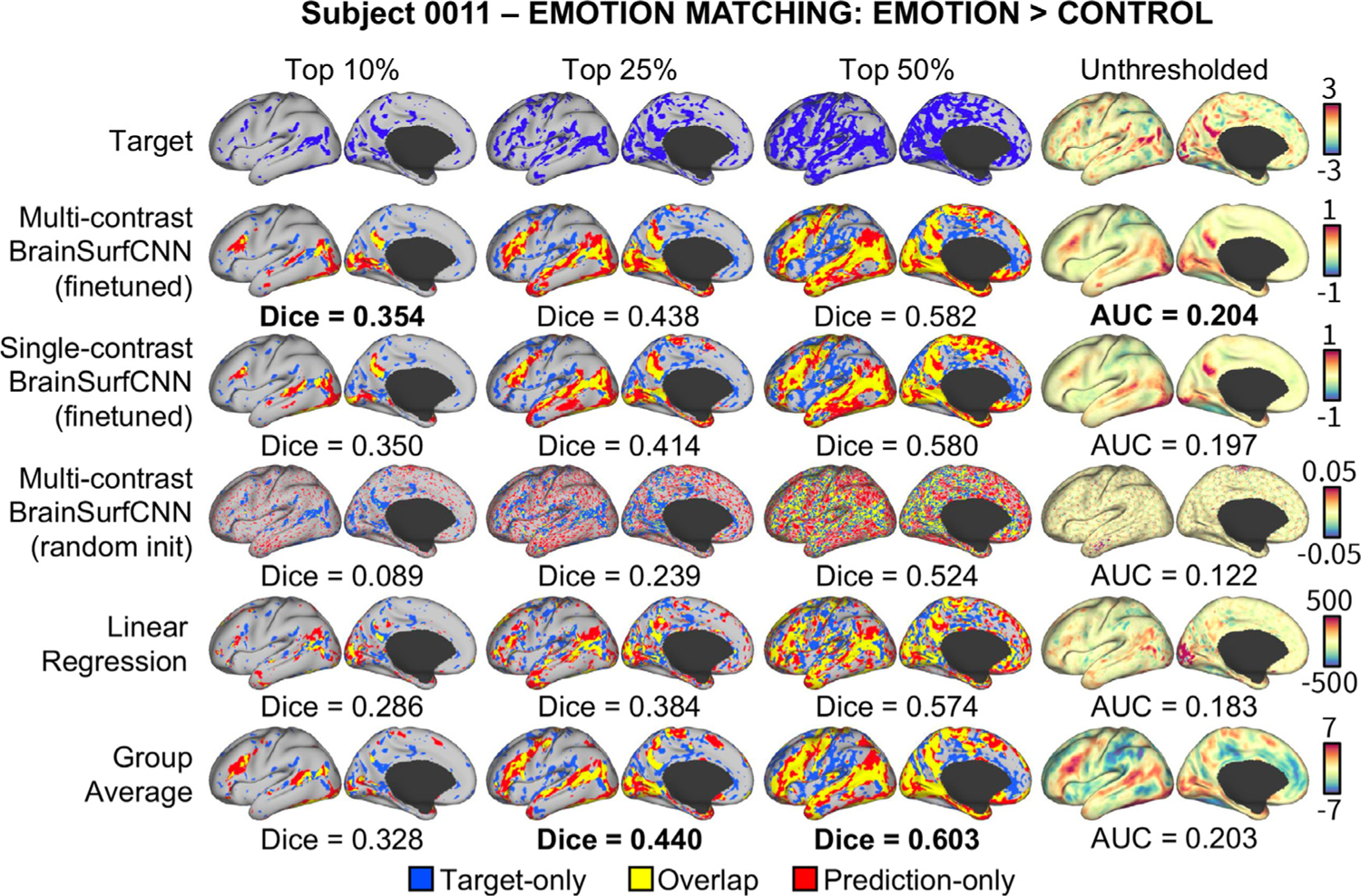
Representative set of model predictions for the “‘Emotion Matching: Emotion > Control” task contrast for a typical subject in Amsterdam PIOP1 dataset. Without finetuning, BrainSurfCNN trained from random initialization failed to capture meaningful patterns of individual subject’s task contrasts. By finetuning on a model pretrained with HCP data (on a different set of task contrasts), BrainSurfCNN models were able to predict individual task contrasts in the Amsterdam PIOP1 dataset.

Fig. 10 shows the Dice scores across all activation thresholds and the overall AUC for the 5 task contrasts in the PIOP1 dataset. When training BrainSurfCNN from scratch - BrainSurfCNN (random init) - the model’s prediction was poor (AUC = 0.128); we hypothesize this is due to the relatively small sample size of the PIOP1. On the other hand, prediction quality was significantly improved by finetuning the model pretrained on the HCP dataset, i.e. BrainSurfCNN (finetuned) (*AUC* = 0. 192; *p* < 10^−5^ for all contrasts). Fig. 11 shows the predicted and measured contrast maps for an average subject and task. The task contrast, “EMOTION MATCHING: EMOTION > CONTROL” has the median target vs. group-average AUC among the 5 PIOP1 task contrasts. For this contrast, subject 0011 has the median AUC with the group-average task contrast. Both Fig. 11 and the Dice graphs in Fig. 10 show that BrainSurfCNN (finetuned) yields significantly higher Dice scores than the group-average contrasts when the most activated or deactivated vertices are considered. This gap, however, shrinks with more liberal thresholds. Overall, the finetuned BrainSurfCNN model’s predictions are on par with the group-average contrasts in terms of AUC (AUC = 0.195), suggesting that the model can capture the overall distribution of the target task contrasts. In this transfer-learning setup, the multi-contrast and single-contrast BrainSurfCNN models (AUC = 0.191) adapted well to the new dataset, resulting in similar predictive performance. However, the multi-contrast setup is more computationally efficient (one model for all outputs) relative to maintaining a separate model for each predictive target.

#### 4.3.2. Amsterdam OpenfMRI PIOP2

**Fig. 12.**
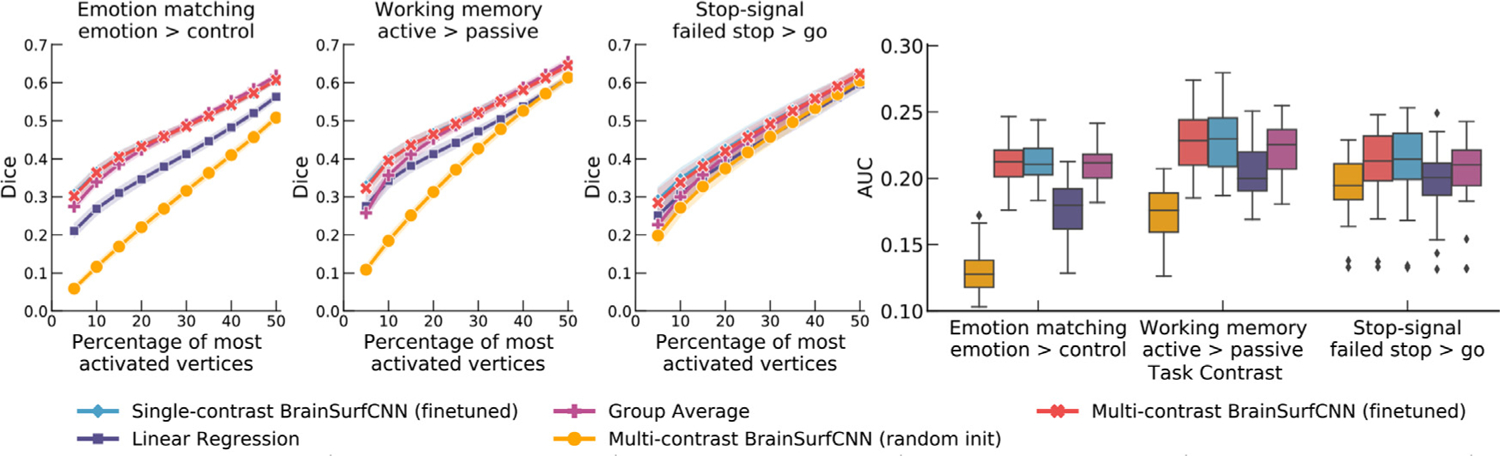
Quality of model prediction (Dice and AUC) for Amsterdam PIOP2 dataset. There is a similar improvement in BrainSurfCNN predictive accuracy via finetuning as for PIOP1 dataset. Compared to the baselines, the finetuned BrainSurfCNN model improves over the linear regression baseline on Dice score across all thresholds, and improves over the group-average reference up to the top 25% most activated vertices.

**Fig. 13.**
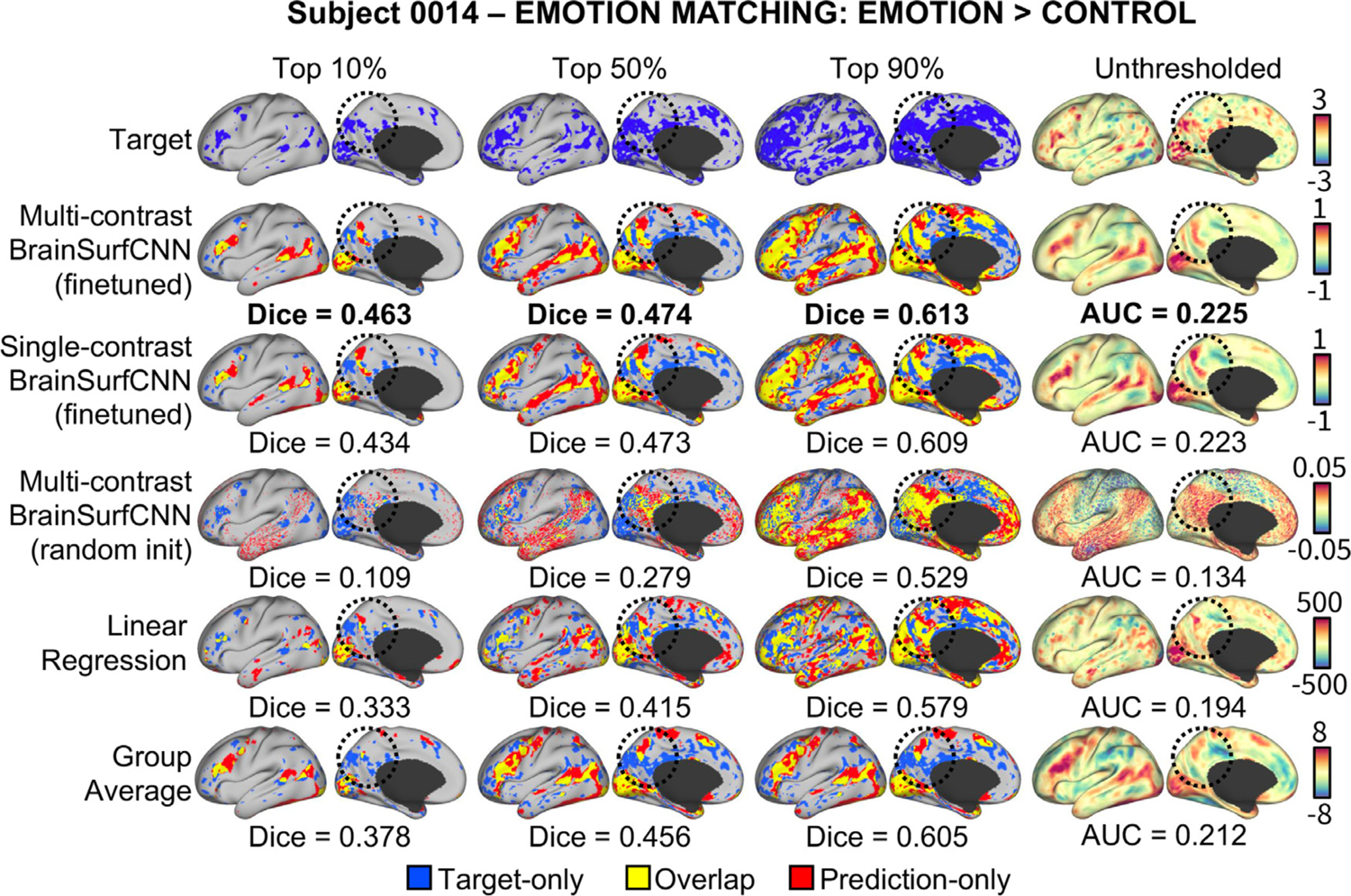
Representative set of model predictions for the Emotion Matching: Emotion > Control of a typical subject in Amsterdam PIOP2 dataset. The finetuned BrainSurfCNN could capture both the gross pattern of the task contrast, as well as the subject-specific details. For example, activation in the posterior medial cortex (circled) was predicted correctly by BrainSurfCNN for the given subject that is not significant in the group-average reference.

Fig. 12 shows the Dice scores across all thresholds and the overall AUC for the 5 task contrasts in the PIOP2 dataset. Similar to our observations above, finetuning the HCP-pretrained BrainSurfCNN model improves predictions over both the linear regression model and the group-average task contrasts. However, in contrast to PIOP1, the BrainSurfCNN trained from random initialization have better predictive performance on PIOP2. Differences in data quality between the two datasets (Snoek et al., 2021) might result in the models having more difficulty to learn on PIOP1 data. Furthermore, the differences in the models’ predictive performances seemingly point to the same trend observed in Fig. 6, i.e., target contrasts that match the group-average more are also better predicted by machine learning models. On average, task contrasts in PIOP2 seem to be more similar to their group-average contrasts (AUC = 0.213) compared to PIOP1 (AUC = 0.196). This is likely because subject-specific deviations from the group average are partly due to noise. Fig. 13 shows the predicted and measured contrast maps of the median subject (0014) and the median task contrast (“EMOTION MATCHING: EMOTION > CONTROL”). Similar to the results on the PIOP1 dataset, BrainSurfCNN has the largest gains over the group-contrast for the most activated vertices.

### 4.4. The shared representation in multi-task learning enables flexible domain adaptation

**Fig. 14.**
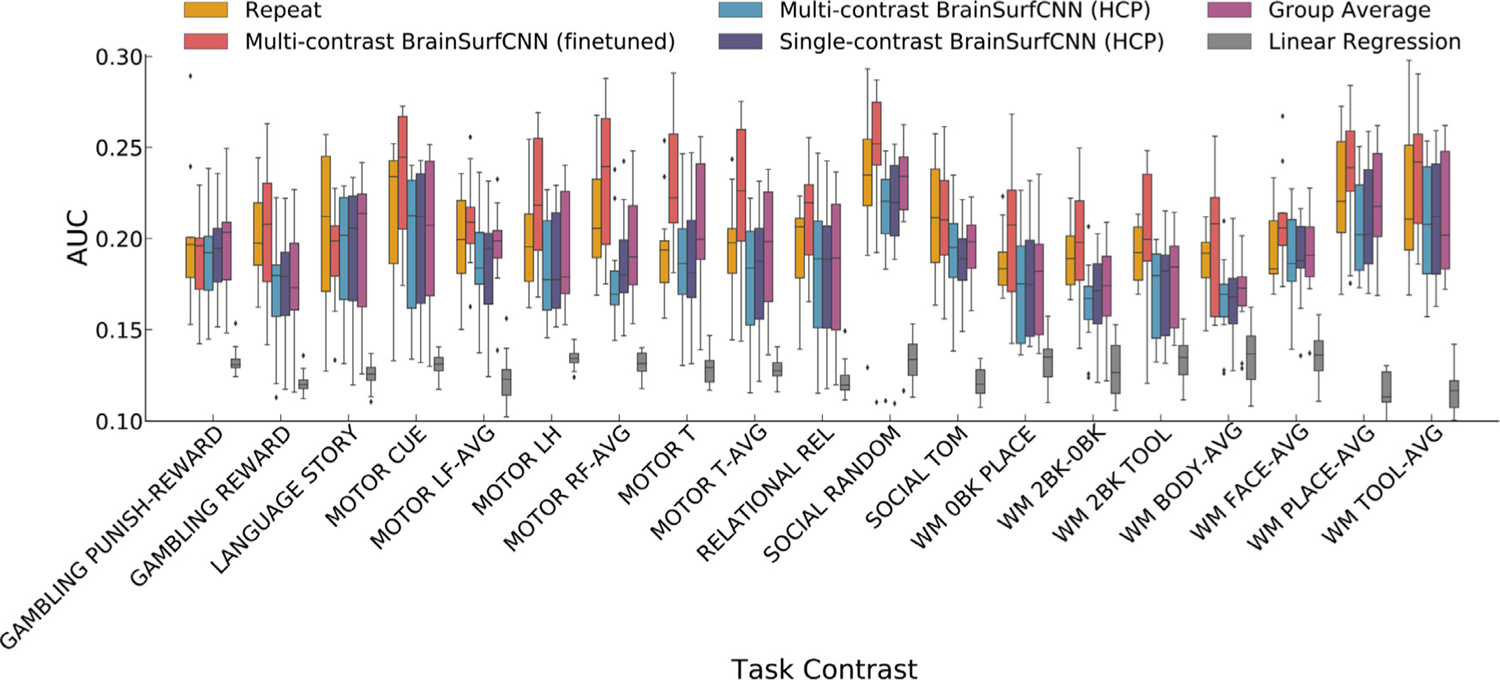
AUC of model predictions on individual-level reliable HCP task contrasts in the IBC dataset. By finetuning the multi-contrast BrainSurfCNN’s backbone on other IBC task contrasts, the model improves its predictive accuracy for the test task contrast over the baselines, as well as the non-finetuned models. Note that only the multi-task learning setting allows such leave-one-task-out training procedure without the model’s access to any training samples of the contrast to be predicted in the IBC dataset.

Fig. 14 shows the AUC scores across 19 individual-level reliable HCP task contrasts in the IBC dataset. Similar to the procedure on the original HCP dataset, reliable contrasts are defined as those whose Dice AUC between the maps derived from the target and repeat scans for HCP task contrasts in the IBC dataset to be larger than the group-average. The finetuned BrainSurfCNN predictions (average AUC = 0.199) are on par with the repeat contrasts (average AUC = 0.187) and the group-average baselines (average AUC = 0.179). They also perform better than the pretrained BrainSurfCNN models (average AUC = 0.173, with p = 4 × 10^−29)^, and better than the linear regression baseline (average AUC = 0.128, with p = 4 × 10^−38)^. The *p*-values were computed from paired 2-tail *t*-test across all subjects and individual-level reliable task contrasts. To reemphasize, the finetuned BrainSurfCNN is not trained on the predicted task contrast from the IBC dataset, but merely benefits from the improved backbone finetuned on the other shared tasks. Fig. 14 shows that indeed the multi-task setup allows learning shared representation that are beneficial across predictive outputs.

**Fig. 15.**
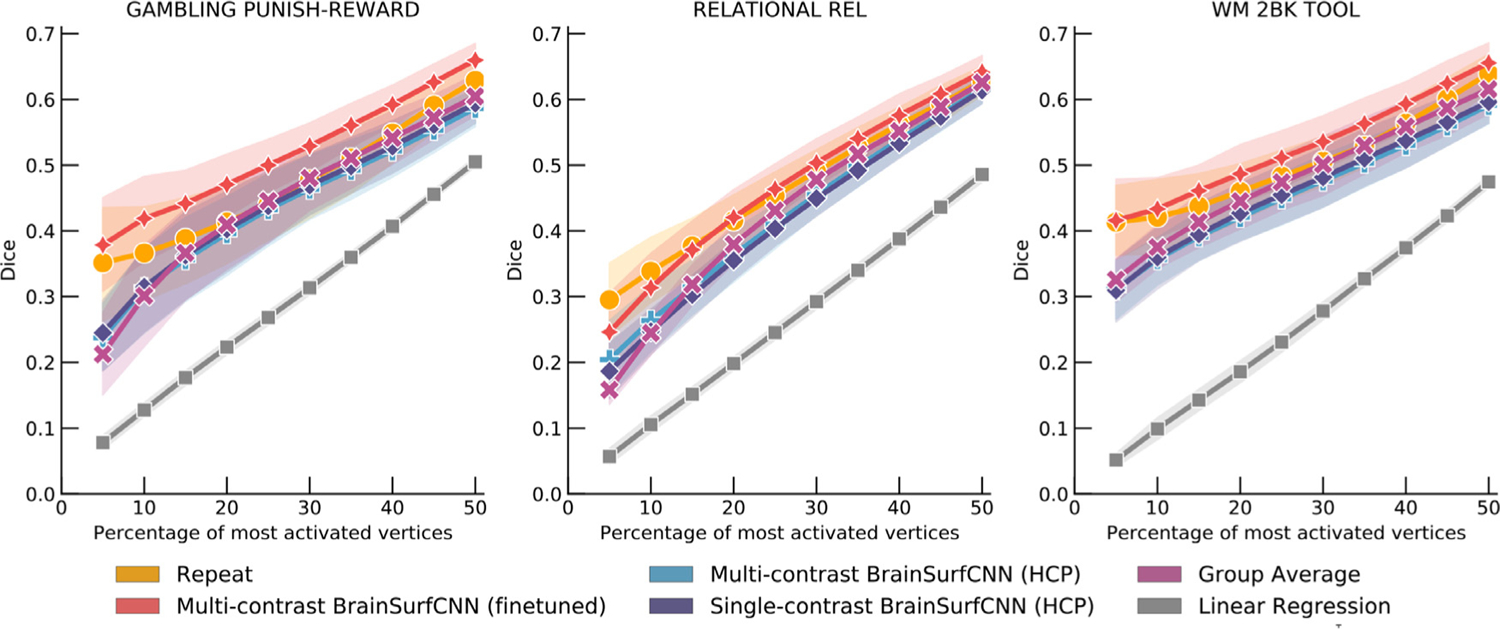
Dice score of model predictions on 3 HCP task contrasts in the IBC dataset at 10th, 50th and 90th average percentile of average AUC among individual-level reliable task contrasts. The BrainSurfCNN finetuned on IBC tasks other than the target task greatly improves the pretrained model’s predictive accuracy (in terms of Dice) across all thresholds of activation.

**Fig. 16.**
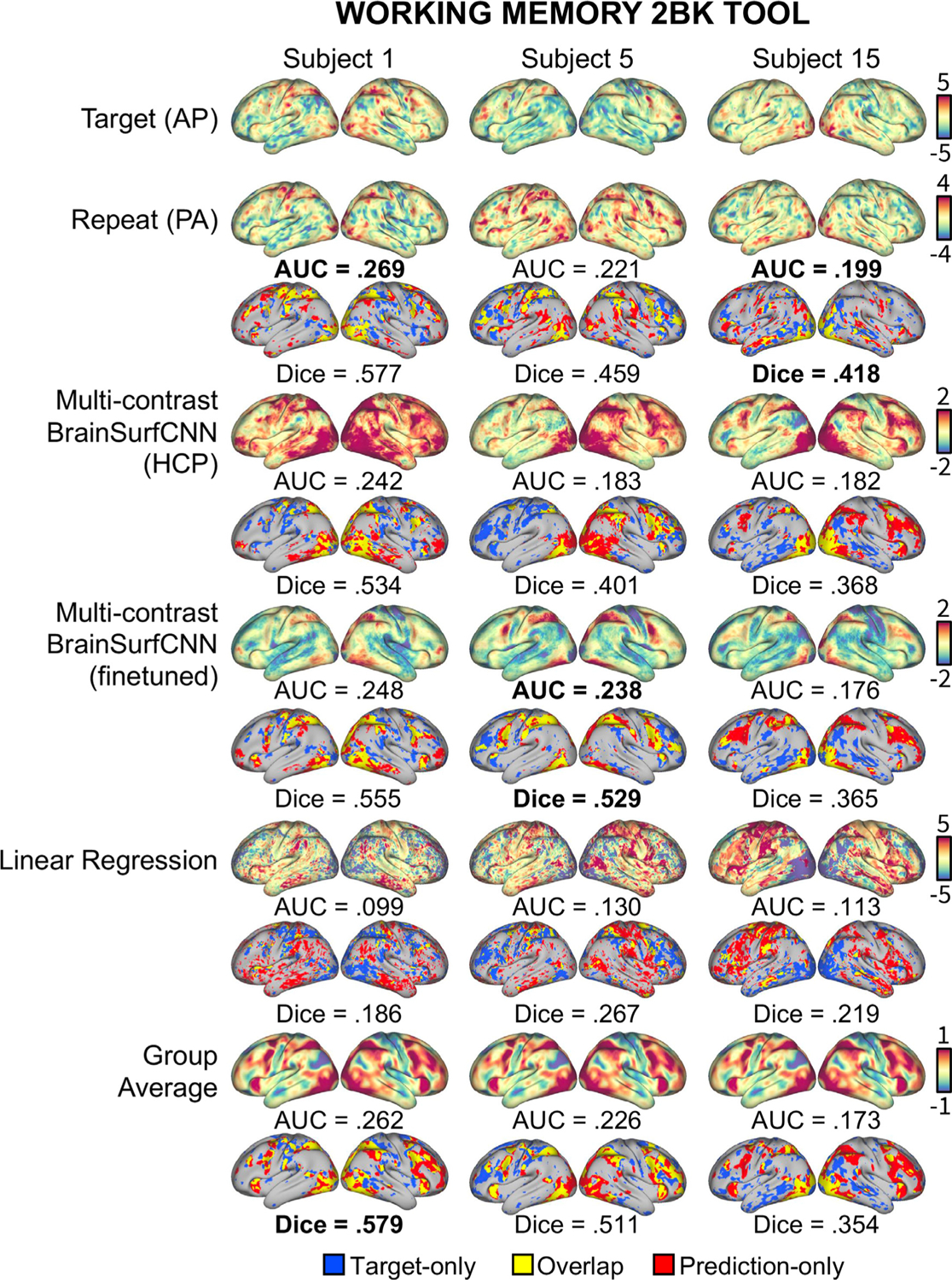
Example model predictions for the “Working memory 2-back tool” contrast of 3 IBC subjects. The finetuned BrainSurfCNN approach the target-repeat reliability in both Dice score for top 25% most activated vertices, and overall AUC across all thresholds.

Fig. 15 shows the Dice scores of 3 contrasts with the highest average reliability AUC scores from 3 unique tasks. The finetuned BrainSurfCNN models improve upon repeat contrasts across all thresholds of activation, except for the top most activated vertices in “RELATIONAL REL” contrast. Fig. 16 shows the target and prediction for the “Working Memory 2BK Toool” task contrast for 3 subjects, including both the unthresholded contrast maps (top rows) and the top 25% most activated vertices (bottom rows). The three subjects, “Subject 1”, “Subject 5”, and “Subject 15” have the 10th, 50th, and 90th percentile “target vs. group-average” AUC among the test subjects, respectively.

## 5. Discussion

Contrasts derived from task-based fMRI have been instrumental for mapping brain responses across subjects and quantifying how they relate to individual traits (McNab and Klingberg, 2008; Mukai et al., 2007; Nijhof and Willems, 2015; Tom et al., 2007; Wang et al., 2019). Task contrasts are also a useful imaging tool for clinical applications (Matthews et al., 2006; Rosazza et al., 2018) such as localizing functional regions in surgical interventions and mapping the impact of lesions. Nonetheless, tfMRI involves meticulous planning and extensive training (Church et al., 2010; Rosazza et al., 2018), and can be prohibitive for some subject groups such as patients or young children. On the other hand, resting-state fMRI is easier to acquire while retaining a rich representation unique to individuals (Amico and Goñi, 2018; Finn et al., 2015; Tian et al., 2021) that overlaps with task-based fMRI (Smith et al., 2009). Therefore, predictive models that can accurately translate a subjects’ resting-state functional connectome to individual-specific task contrasts might unlock new venues of applications where only rsfMRI is available, especially in personalized prediction of phenotypes or diagnosis where inter-subject variation is of foremost interest (Castellanos et al., 2013; Finn and Constable, 2016; Fornito and Bullmore, 2011). Furthermore, as several works have pointed out strong correlations between intrinsic functional connectivity measured by rsfMRI and neural phenotypes like behavioral traits (Liégeois et al., 2019), or psychological risk factors (Cherkassky et al., 2006; Tian et al., 2006), we surmise that future extension of BrainSurfCNN might also be applicable for such predictive objectives. Another interesting angle might be combining BrainSurfCNN with probing tools, such as (Dabkowski and Gal, 2017), or causal inference (Pearl, 2009; Schölkopf et al., 2021), to go beyond black-box prediction and unlock new insights linking functional connectivity and brain activity durng tasks or behavioral traits. Lastly, we have focused solely on healthy subjects in this work, but BrainSurfCNN can potentially be applied to patient populations to investigate the effect of disease on task-evoked brain activity, similar to Jones et al. (2017).

Surface-based preprocessing and analytical methods are increasingly popular in fMRI as they have shown promising results in improving registration, smoothing and functional localization (Coalson et al., 2018). In contrast, surfaced-based predictive models for neuroimaging data are not well studied. Previous works have used linear regression models to predict vertex-wise brain response for a given task contrast from resting-state functional connectomes (Cole et al., 2016; Tavor et al., 2016). In Dohmatob et al. (2021), local gradients estimated from rsfMRI were used as input features, but the model also made use of a parcel-wise linear regression to predict task activation. Other neural network based approaches have used heavily preprocessed inputs, such as low-dimensional ROI-based functional connectivity matrices (Kawahara et al., 2017) or population graphs (Parisot et al., 2017), which often reduce otherwise rich data into summary metrics and greatly diminish spatial resolution. In a different approach using graph neural networks, (Zhang et al., 2021) predicts brain states from fMRI timeseries mapped on to a brain graph. A notable exception that is closely related to our approach but not focused on functional imaging, is (Zhao et al., 2019), which is a neural network operating on the spherical representation of structural brain images and uses a different approximation for the convolutional operation by limiting the kernel to immediate neighbors of each vertex. Furthermore, although we made use of the surface-based functional connectivity representation proposed in Tavor et al. (2016) and Cole et al. (2016) as it suits our goal of predicting functional localization on the cortical surface, other representation of rsfMRI data would suit other analytical purpose or preprocessing pipeline (Khosla et al., 2019b). Such endeavors are beyond the scope of this work and will be left for future work.

In this paper, we present BrainSurfCNN, a new neural network model for predicting task activation in individual subjects. We demonstrate that BrainSurfCNN can well predict subject-specific patterns in tfMRI task contrasts from the HCP dataset. We also presented that using two different transfer learning strategies, the pretrained BrainSurfCNN can generalize to some extent to new datasets or new task contrasts that otherwise have limited training samples available. By using a multi-task learning framework, the model was encouraged to learn a representation shared across multiple tasks (Baxter, 2000; Caruana, 1997; Maurer et al., 2016; Mensch et al., 2017), which allows the model to make accurate prediction on task contrasts that were not yet seen during training.

We believe that our proposed approach can be improved via future research. Firstly, there are likely architectural choices that can afford a performance boost. For example, if the data are registered to a common brain template, this additional information can potentially be exploited by a neural network via the coordinate-aware convolution operation of Liu et al. (2018). Secondly, capturing inter-individual variation in model prediction might require new strategies. In our experiments, we observed that training on a single loss between a subject’s predicted output and measured contrast seems to push the model’s prediction toward the group-average, possibly because inter-subject variation is significantly smaller than the average signal magnitude. Curiously, we found that training on individual residuals from the group average, i.e. difference between a subject’s task contrast and the group average, is not effective (not reported). By introducing a contrastive loss that maximizes inter-subject differences, BrainSurfCNN could enhance features specific to each individual subject (shown in our ablation results of Supplemental Table 1). Nonetheless, we found that contrastive loss is not effective when finetuning on smaller datasets, possibly because a more careful search for the right loss margins is needed. Finally, the effect of different preprocessing choices on the downstream predictive task can be further explored. While we opted for minimal preprocessing, there is evidence that appropriate denoising can improve predictive quality (Kelly et al., 2012). Furthermore, the choice of ROIs affects input functional connectivity (Salehi et al., 2020; Thirion et al., 2014) and might affect the downstream prediction task. In our experiments, we had a limit of 50 ROIs due to computational resource constraints. We are working on improving BrainSurfCNN’s efficiency, which can allow using input connectomes with more number of features that can better capture subject-specific variability. To facilitate future studies, the source code for our models and analysis are publicly available at https://github.com/sabunculab/brainsurfcnn.

## Supplementary Material

Supplementary Material

## Data Availability

All code that is implemented and used for the present article is made available at https://github.com/sabunculab/brainsurfcnn and will be maintained and supported through this website. All data used in this article is publicly available and the authors are not part of the consortia that have collected these data. Corresponding references where further information can be found, are made available in the manuscript.
