# Supplementary material for: Predicting individual task contrasts from resting-state functional connectivity using a surface-based convolutional network
Source: Neuroimage. Author manuscript; Available in PMC 2023 May 3. (PMC10155599; doi:10.1016/j.neuroimage.2021.118849)
Supplement: Supplementary Material [file NIHMS1892335-supplement-Supplementary_Material.pdf]

Supplemental Materials for “Predicting Individual Task Contrasts From Resting-state Functional Connectivity using a Surface-based Convolutional Network’

1. Supplemental Figures

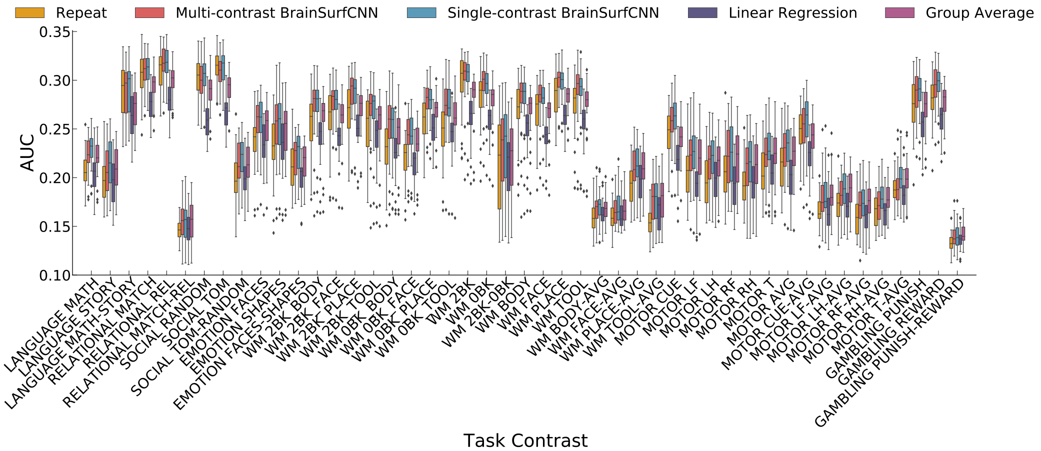

Supplemental Figure 1: Area under the Dice scores curve between target and predicted (or reference) thresholded activation maps for all 47 HCP task contrasts.

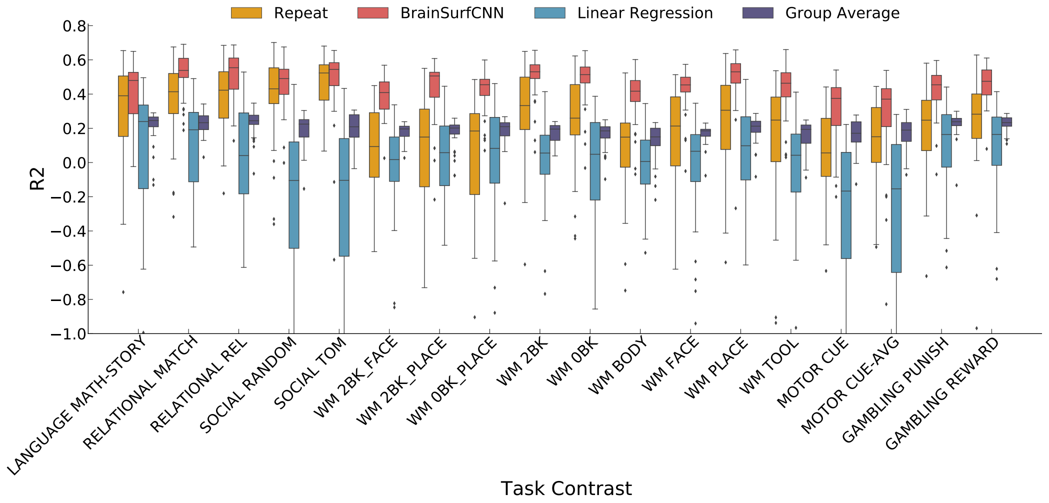

Supplemental Figure 2: BrainSurfCNN prediction is better than the linear regression prediction and group-average contrast map while approaching reliability of the task contrast (measured by the correspondence with the repeat task contrast map) across the most reliable HCP task contrasts (whose target-repeat reliability R^2 is higher than 0). Quality of prediction is measured as the explained variance (R^2 score) between target and predicted activation maps. Supplemental Table 6 includes the average R^2 of each model across the 18 reliable HCP task contrasts. Supplemental Table 7 shows the p-values from paired 2-tail t-test of R^2 scores between models.

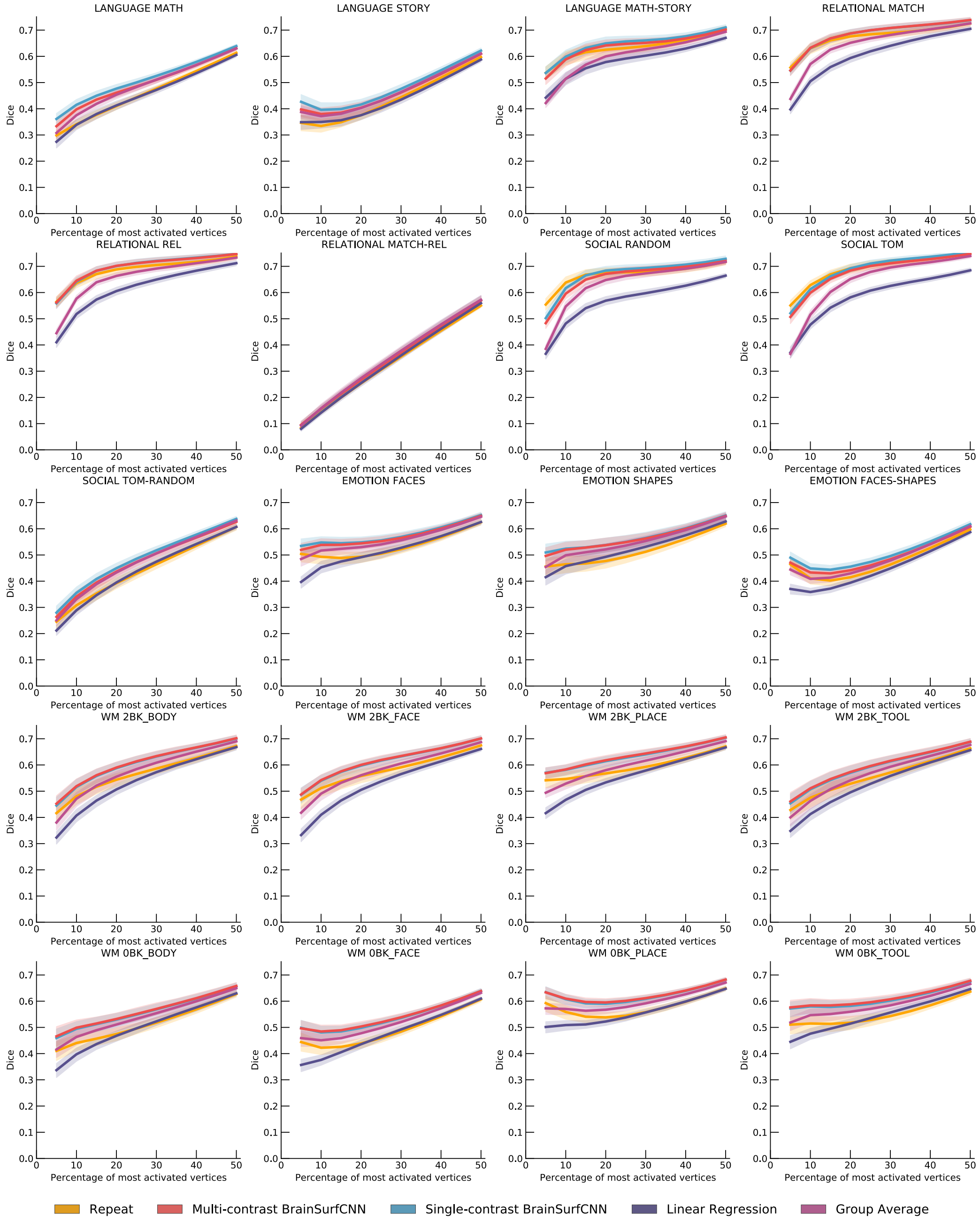

Supplemental Figure 3: Dice scores of overlap between target and predicted (or reference) activation maps for all 47 HCP task contrasts over thresholds ranged between 5% to 50% of most activated vertices (part 1)

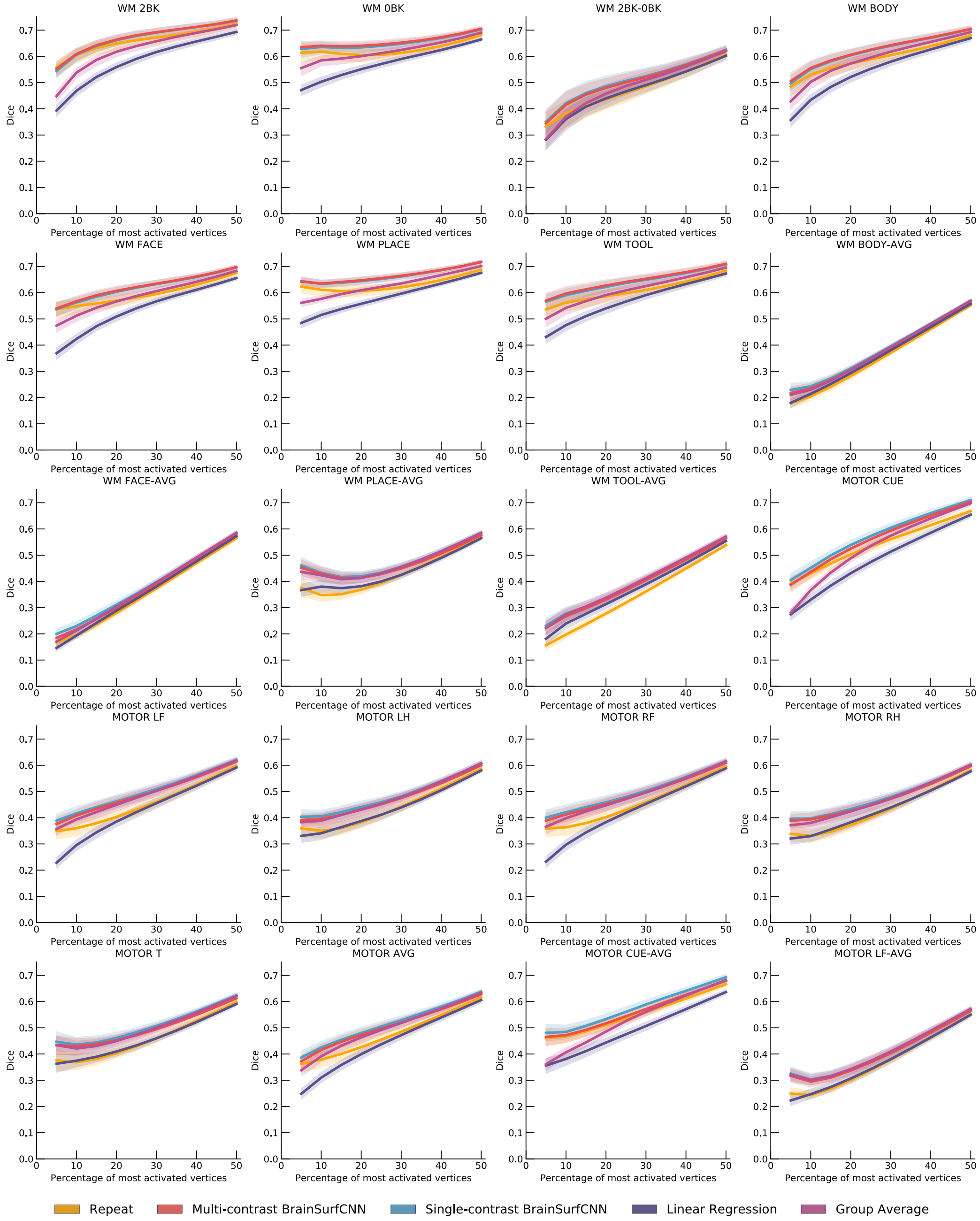

Supplemental Figure 4: Dice scores of overlap between target and predicted (or reference) activation maps for all 47 HCP task contrasts over thresholds ranged between 5% to 50% of most activated vertices (part 2).

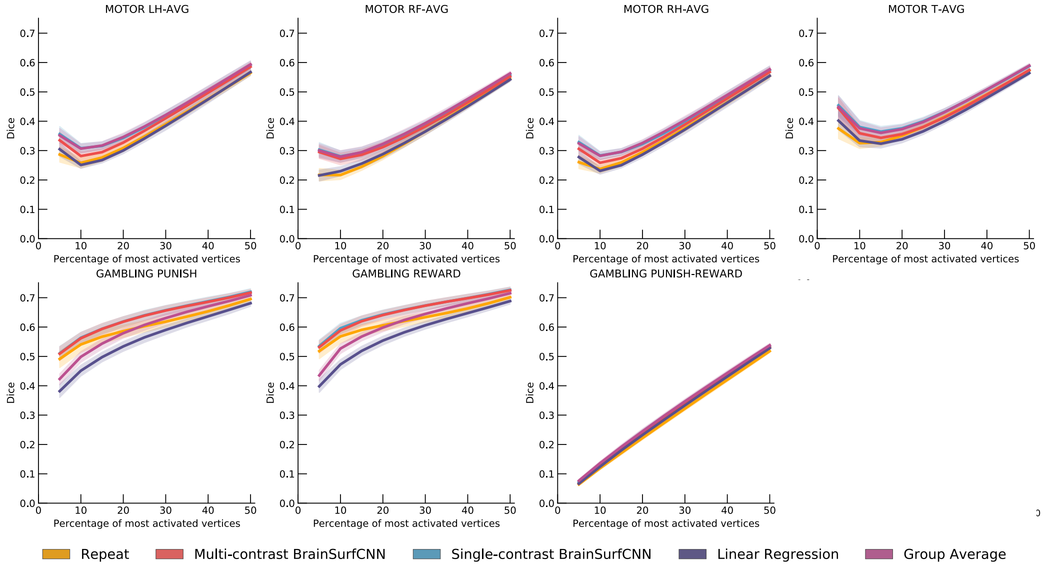

Supplemental Figure 5: Dice scores of overlap between target and predicted (or reference) activation maps for all 47 HCP task contrasts over thresholds ranged between 5% to 50% of most activated vertices (part 3).

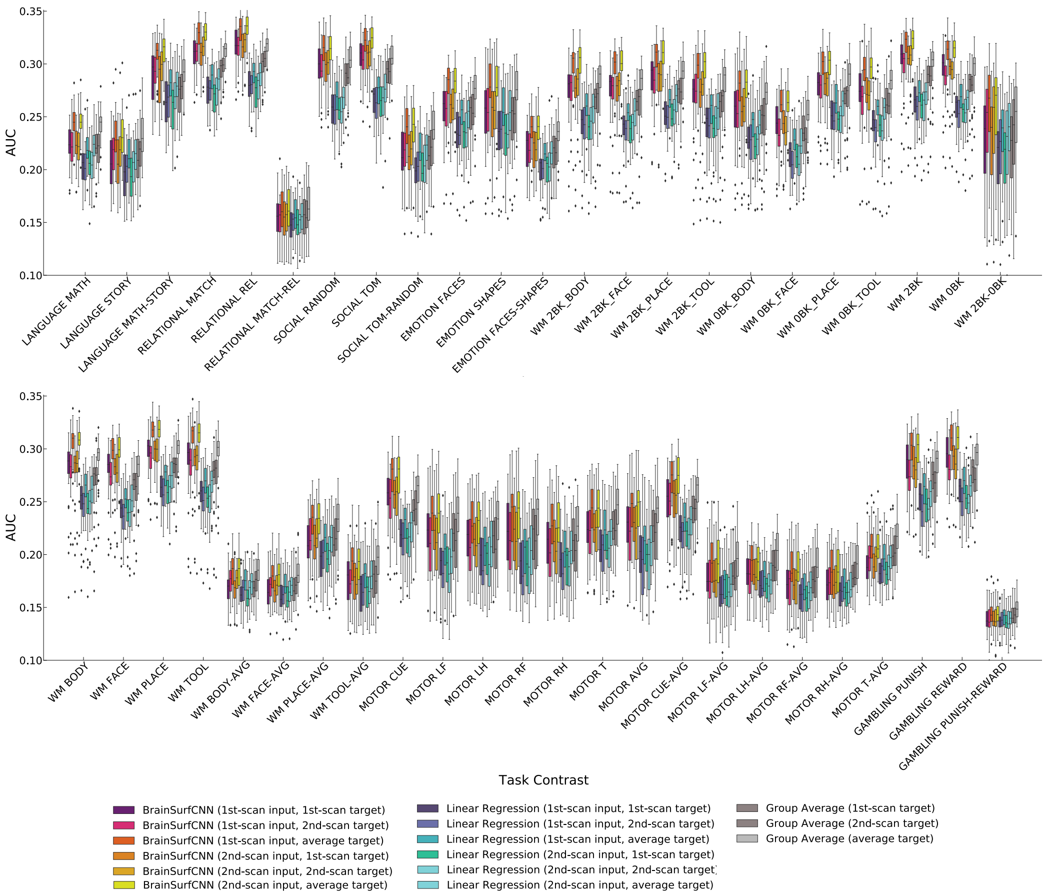

Supplemental Figure 6: predictive accuracy measured by Dice AUC between predicted contrast maps and target tfMRI task contrasts from the *first* scan, *repeat* (second) scan, and *average* target tfMRI task contrasts (average of the contrasts from the first and second scan) of 39 HCP test subjects. BrainSufCNN and linear regression models both use rsfMRI input from the test subjects *first* or *repeat* (second) scan.

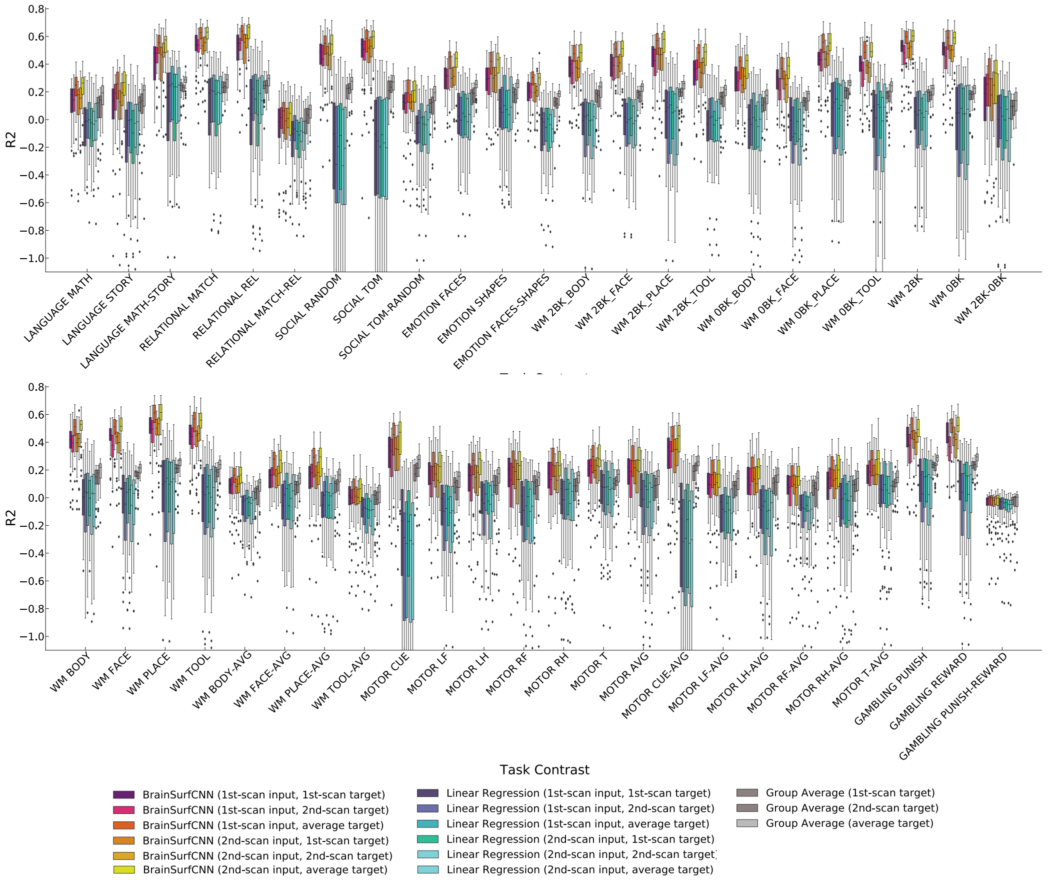

Supplemental Figure 7: predictive accuracy measured by R2 score between predicted contrast maps and target tfMRI task contrasts from the *first* scan, *repeat* (second) scan, and *average* target tfMRI task contrasts (average of the contrasts from the first and second scan) of 39 HCP test subjects. BrainSufCNN and linear regression models both use rsfMRI input from the test subjects first or repeat (second) scan.

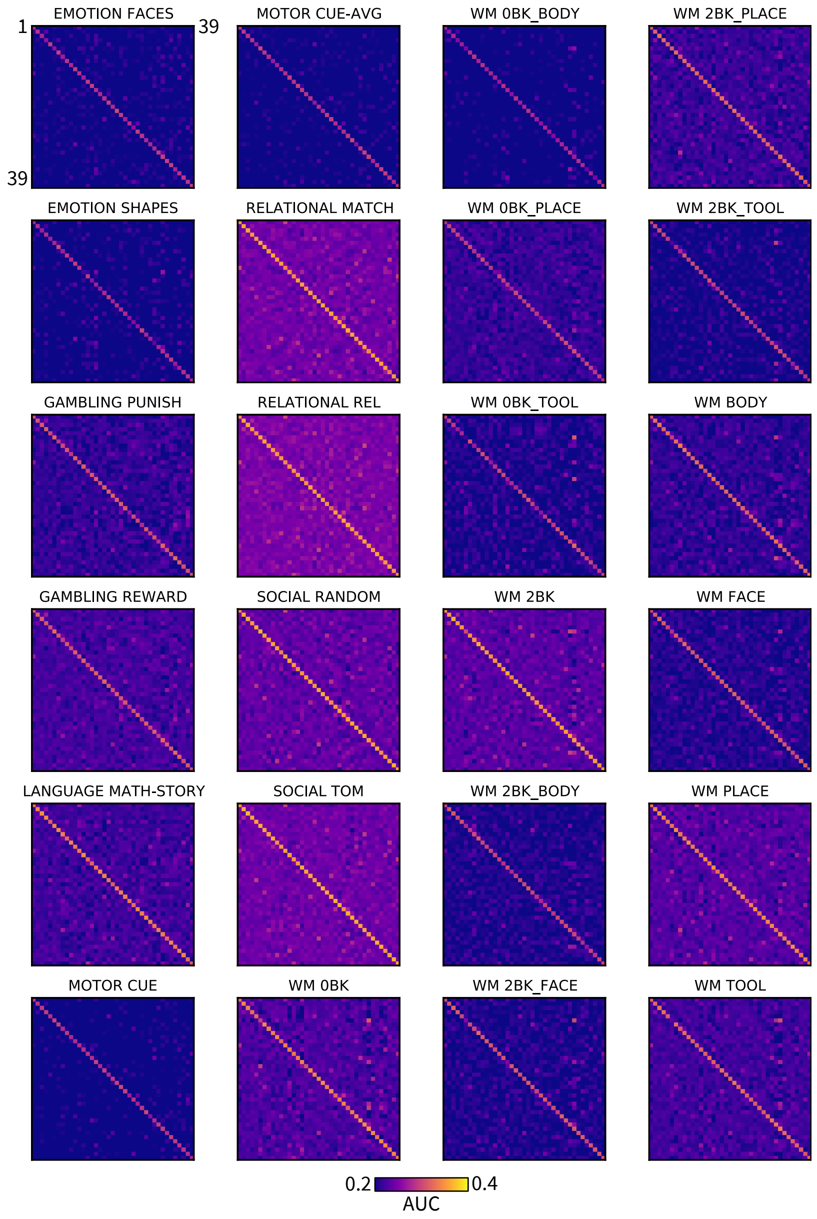

Supplemental Figure 8: Dice AUC between individual tfMRI-derived target contrast maps and task contrasts derived from the repeat scan of all 39 HCP test subjects for all 24 individual-level reliable HCP task contrasts. Each row corresponds to a target contrast map and each column corresponds to a subject's task contrast estimated from the repeat scan.

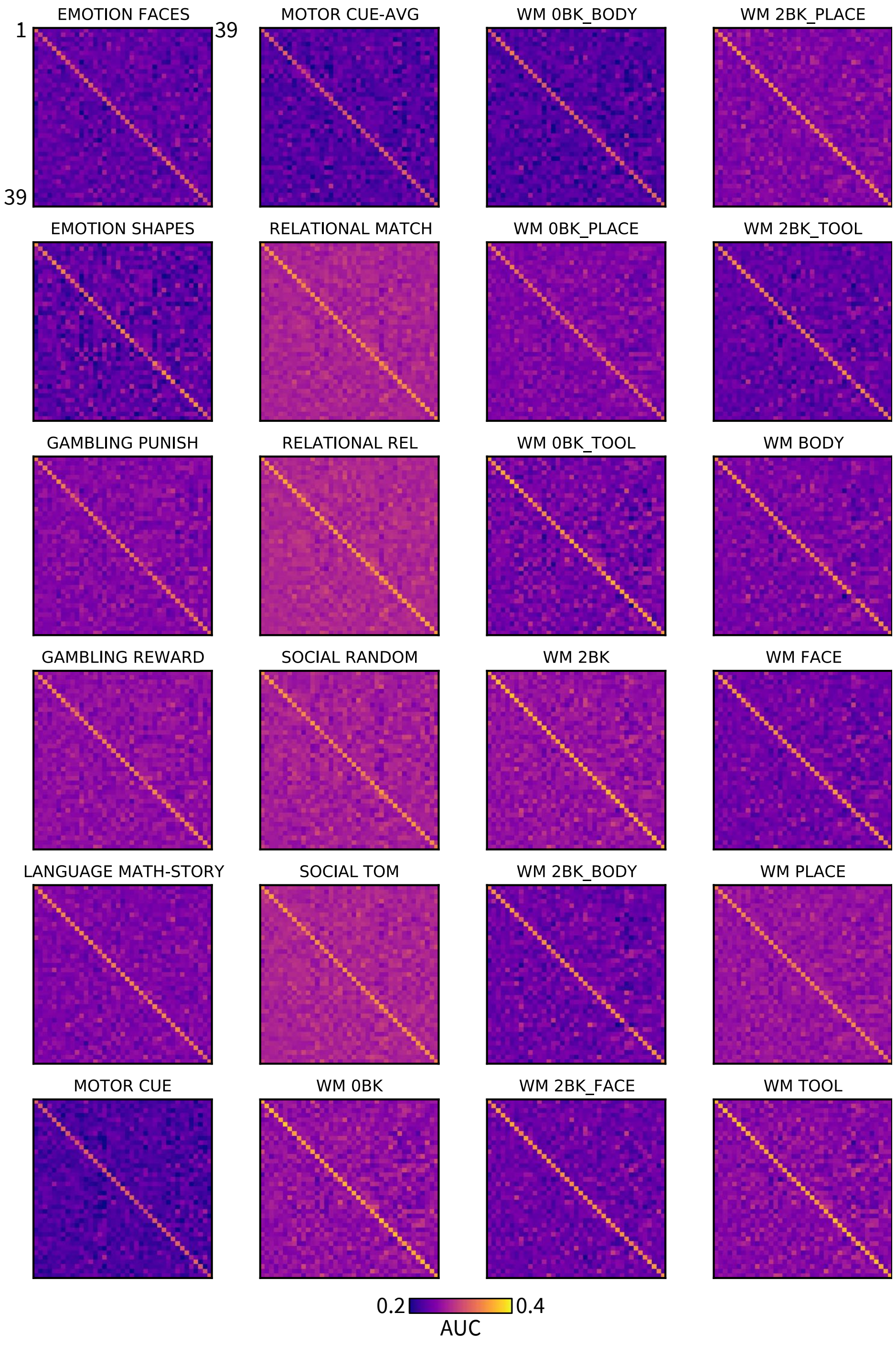

Supplemental Figure 9: Dice AUC between individual task contrast maps predicted by the multi-contrast BrainSurfCNN and the tfMRI-derived target contrast maps of all 39 HCP test subjects for all 24 individual-level reliable HCP task contrasts. Each row corresponds to a target contrast map and each column corresponds to a predicted contrast map.

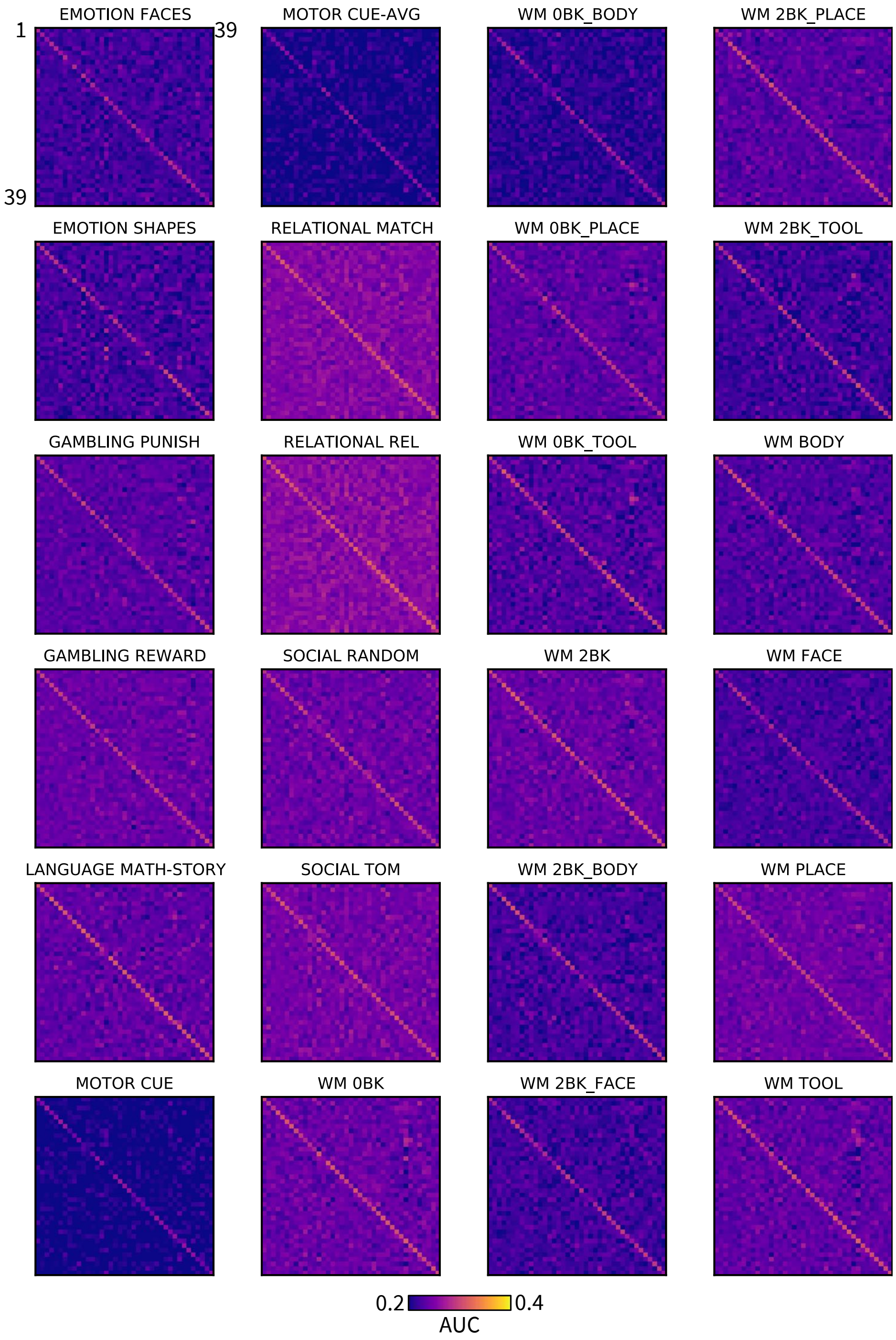

Supplemental Figure 10: Dice AUC between individual task contrast maps predicted by the linear regression model and the tfMRI-derived target contrast maps of all 39 HCP test subjects for all 24 individual-level reliable HCP task contrasts. Each row corresponds to a target contrast map and each column corresponds to a predicted contrast map.

2. Supplemental Tables

Model	AUC with same subject's target contrasts	AUC with same subject's minus AUC with other subjects' target contrasts
BrainSurfCNN	0.187 ± 0.049	0.025 ± 0.018
BrainSurfCNN without contrastive loss	0.179 ± 0.047	0.014 ± 0.010
BrainSurfCNN without skip connections	0.147 ± 0.046	0.006 ± 0.005

Supplemental Table 1: Ablation study of BrainSurfCNN architecture and training. The three models used for comparison are (1) full BrainSurfCNN model and training model as described in Methods and Materials section; (2) BrainSurfCNN trained only on mean squared error trained with the same number of epochs as the full model (BrainSurfCNN without contrastive loss) (3) BrainSurfCNN with no skip connection, which is equivalent to an auto encoder architecture, but doubled in number of channels per intermediate layers to retain comparable number of parameters to the full model. The models were evaluated on the HCP test set across all 47 task contrasts using Dice AUC metrics using the checkpoint with the lowest error on the validation set. The ablation study shows that skip connections contribute significantly to BrainSurfCNN performance. Furthermore, the contrastive loss both improves the model's prediction AUC with the same subject's target contrasts and make the prediction more different from other subjects' target contrasts in terms of Dice AUC.

Task	Contrast	Abbreviation	Present in IBC
Language	Math	LANGUAGE MATH	Yes
	Story	LANGUAGE STORY	Yes
	Math - story	LANGUAGE MATH-STORY	Yes
Relational Processing	Relation	RELATIONAL REL	Yes
	Match	RELATIONAL MATCH	Yes
	Match - relation	RELATIONAL MATCH-REL	
Social Cognition	Mental interaction	SOCIAL TOM	Yes
	Random interaction	SOCIAL RANDOM	Yes
	Mental - random interaction	TOM-RANDOM	Yes
Emotional Processing	Emotional faces	EMOTION FACES	Yes
	Shapes	EMOTION SHAPES	Yes
	Emotional faces-shapes	EMOTION FACES-SHAPES	Yes
Gambling	Reward	GAMBLING REWARD	Yes
	Punish	GAMBLING PUNISH	Yes
	Punish - reward	GAMBLING PUNISH-REWARD	Yes

Supplemental Table 2: Task contrasts from the Human Connectome Project (HCP). Details of the imaging and preprocessing protocols are available in [2]. The last column indicates if the task contrasts are also present in the IBC dataset.

Task	Contrast	Abbreviation	Present in IBC
Working Memory	0-back body	WM 0BK_BODY	Yes
	0-back face	WM 0BK_FACE	Yes
	0-back place	WM 0BK_PLACE	Yes
	0-back tool	WM 0BK_TOOL	Yes
	2-back body	WM 2BK_BODY	Yes
	2-back face	WM 2BK_FACE	Yes
	2-back place	WM 2BK_PLACE	Yes
	2-back tool	WM 2BK_TOOL	Yes
	2-back	WM 2BK	
	0-back	WM 0BK	
	2-back - 0-back	2BK-0BK	Yes
	Body	WM BODY	
	Face	WM FACE	
	Place	WM PLACE	
	Tool	WM TOOL	
	Body - average	WM BODY-AVG	Yes
	Face - average	WM FACE-AVG	Yes
	Place - average	PLACE-AVG	Yes
	Tool - average	TOOL-AVG	Yes
Motor	Cue	MOTOR CUE	
	Left foot	MOTOR LF	Yes
	Left hand	MOTOR LH	Yes
	Right foot	MOTOR RF	Yes
	Right hand	MOTOR RH	Yes
	Tongue	MOTOR T	Yes
	Average	MOTOR AVG	
	Cue - average	MOTOR CUE-AVG	
	Left foot - average	MOTOR LF-AVG	Yes
	Left hand - average	MOTOR LH-AVG	Yes
	Right foot - average	MOTOR RF-AVG	Yes
	Right hand - average	MOTOR RH-AVG	Yes
	Tongue - average	MOTOR T-AVG	Yes

Supplemental Table 3: Task contrasts from the Human Connectome Project (HCP) (continued). Details of the imaging and preprocessing protocols are available in [2]. The last column indicates if the task contrasts are also present in the IBC dataset.

HCP Task Contrast	Dice AUC			
	Multi-contrast BrainSurfCNN	Repeat	Linear regression	Group average
LANGUAGE MATH-STORY	**0.30**	**0.30**	0.28	0.29
RELATIONAL MATCH	**0.31**	**0.31**	0.29	0.29
RELATIONAL REL	**0.34**	0.33	0.31	0.32
SOCIAL RANDOM	**0.32**	**0.32**	0.28	0.30
SOCIAL TOM	0.31	**0.33**	0.27	0.30
EMOTION FACES	**0.27**	0.24	0.26	0.26
EMOTION SHAPES .	**0.28**	0.24	0.27	0.27
WM 2BK BODY	**0.27**	0.26	0.25	0.26
WM 2BK FACE	**0.28**	**0.28**	0.27	0.26
WM 2BK PLACE	**0.30**	0.29	0.29	0.29
WM 2BK TOOL .	**0.27**	0.26	0.26	0.26
WM 0BK BODY	**0.24**	0.23	0.23	0.23
WM 0BK PLACE	**0.29**	0.27	0.27	0.28
WM 0BK TOOL	**0.24**	0.23	0.23	**0.24**
WM 2BK	**0.31**	0.30	0.29	0.29
WM 0BK	**0.28**	**0.28**	0.27	0.27
WM BODY	**0.28**	0.26	0.26	0.27
WM FACE	**0.28**	0.28	0.27	0.27
WM PLACE	**0.31**	0.30	0.29	0.29
WM TOOL	**0.29**	0.28	0.26	0.27
MOTOR CUE	**0.26**	**0.26**	0.22	0.25
MOTOR CUE-AVG	**0.27**	0.26	0.24	0.26
GAMBLING PUNISH	**0.31**	0.29	0.28	0.29
GAMBLING REWARD	**0.30**	0.29	0.28	0.29

Supplemental Table 4: Dice AUC between predicted and target task tfMRI activation maps of 24 reliable HCP task contrasts. The scores in bold denote the highest values for a given task contrast.

HCP Task Contrast	Paired 2-tail t-test p-value			
	BrainSurfCNN vs. Repeat	BrainSurfCNN vs. Linear regression	BrainSurfCNN vs. Group average	Linear regression vs. Group average
LANGUAGE MATH-STORY	0.35	9×10^{-19}	5×10^{-14}	3×10^{-5}
RELATIONAL MATCH	0.02	10^{-25}	4×10^{-19}	2×10^{-15}
RELATIONAL REL	0.02	7×10^{-26}	2×10^{-17}	10^{-14}
SOCIAL RANDOM	0.09	5×10^{-26}	4×10^{-10}	7×10^{-21}
SOCIAL TOM	0.04	8×10^{-28}	10^{-17}	10^{-19}
EMOTION FACES	2×10^{-10}	10^{-19}	4×10^{-9}	2×10^{-14}
EMOTION SHAPES	2×10^{-10}	8×10^{-15}	3×10^{-7}	2×10^{-10}
WM 2BK BODY	2×10^{-8}	10^{-19}	2×10^{-14}	4×10^{-13}
WM 2BK FACE	9×10^{-5}	10^{-22}	2×10^{-17}	2×10^{-14}
WM 2BK PLACE	2×10^{-8}	4×10^{-21}	5×10^{-18}	6×10^{-14}
WM 2BK TOOL	5×10^{-6}	2×10^{-17}	2×10^{-15}	2×10^{-11}
WM 0BK BODY	10^{-8}	10^{-17}	2×10^{-12}	3×10^{-14}
WM 0BK PLACE	3×10^{-8}	3×10^{-21}	10^{-13}	5×10^{-14}
WM 0BK TOOL	10^{-10}	2×10^{-21}	3×10^{-15}	6×10^{-14}
WM 2BK	0.06	2×10^{-22}	3×10^{-19}	2×10^{-13}
WM 0BK	5×10^{-5}	2×10^{-21}	4×10^{-17}	8×10^{-14}
WM BODY	2×10^{-5}	210^{-19}	6×10^{-15}	6×10^{-14}
WM FACE	5×10^{-5}	4×10^{-22}	2×10^{-17}	10^{-14}
WM PLACE	2×10^{-6}	2×10^{-23}	4×10^{-19}	3×10^{-15}
WM TOOL	2×10^{-5}	10^{-22}	10^{-17}	3×10^{-13}
MOTOR CUE	10^{-4}	3×10^{-22}	2×10^{-13}	8×10^{-14}
MOTOR CUE-AVG	0.1	2×10^{-19}	4×10^{-10}	2×10^{-12}
GAMBLING PUNISH	10^{-4}	10^{-20}	10^{-17}	10^{-11}
GAMBLING REWARD	5×10^{-5}	3×10^{-20}	5×10^{-10}	7×10^{-12}

Supplemental Table 5: p-value from paired 2-tail t-test of Dice AUC scores between predicted and target task fMRI activation maps. The R2 scores are computed across 18 reliable HCP task contrasts of 39 test subjects.

HCP Task Contrast	Whole brain R^2 score			
	Multi-contrast BrainSurfCNN	Repeat	Linear regression	Group average
LANGUAGE MATH-STORY	**0.42**	0.29	0.09	0.21
RELATIONAL MATCH	**0.52**	0.36	0.02	0.23
RELATIONAL REL	**0.52**	0.37	0.00	0.23
SOCIAL RANDOM	**0.45**	0.38	-0.26	0.19
SOCIAL TOM	**0.48**	0.43	-0.33	0.20
WM 2BK FACE	0.40	**0.42**	-0.02	0.18
WM 2BK PLACE	**0.43**	0.07	-0.01	0.17
WM 0BK PLACE	**0.42**	0.05	0.00	0.18
WM 2BK .	**0.50**	0.30	-0.01	0.17
WM 0BK	**0.48**	0.25	0.00	0.16
WM BODY	**0.38**	0.09	-0.03	0.16
WM FACE	**0.43**	0.14	-0.03	0.13
WM PLACE	**0.50**	0.22	0.00	0.17
WM TOOL	**0.43**	0.14	-0.01	0.20
MOTOR CUE	**0.31**	0.00	-0.39	0.16
MOTOR CUE-AVG	**0.27**	0.08	-0.40	0.17
GAMBLING PUNISH	**0.43**	0.20	0.09	0.22
GAMBLING REWARD	**0.46**	0.25	0.09	0.23

Supplemental Table 6: R^2 scores between predicted and target task tfMRI activation maps of 18 HCP task contrasts whose target-repeat reliability R^2 is higher than 0. The scores in bold denote the highest values for a given task contrast.

HCP Task Contrast	Paired 2-tail t-test p-value			
	BrainSurfCNN vs. Repeat	BrainSurfCNN vs. Linear regression	BrainSurfCNN vs. Group average	Linear regression vs. Group average
LANGUAGE MATH-STORY	5×10^{-4}	4×10^{-10}	2×10^{-11}	3×10^{-2}
RELATIONAL MATCH	4×10^{-8}	9×10^{-8}	3×10^{-16}	2×10^{-2}
RELATIONAL REL	1×10^{-6}	10^{-9}	5×10^{-13}	5×10^{-3}
SOCIAL RANDOM	0.02	5×10^{-12}	1.7×10^{-12}	3×10^{-6}
SOCIAL TOM	0.10	9×10^{-10}	3×10^{-9}	10^{-4}
WM 2BK FACE	3×10^{-8}	2×10^{-13}	2×10^{-16}	3×10^{-5}
WM 2BK PLACE	10^{-10}	5×10^{-12}	7×10^{-16}	9×10^{-4}
WM 0BK PLACE	10^{-10}	3×10^{-10}	8×10^{-16}	3×10^{-3}
WM 2BK	3×10^{-6}	10^{-12}	4×10^{-19}	2×10^{-3}
WM 0BK	$\times 10^{-9}$	10^{-11}	5×10^{-19}	6×10^{-3}
WM BODY	3×10^{-9}	7×10^{-14}	3×10^{-15}	3×10^{-4}
WM FACE	3×10^{-7}	3×10^{-12}	2×10^{-18}	2×10^{-4}
WM PLACE	4×10^{-9}	2×10^{-11}	7×10^{-18}	3×10^{-3}
WM TOOL	3×10^{-7}	5×10^{-12}	4×10^{-1}	4×10^{-3}
MOTOR CUE	3×10^{-8}	7×10^{-11}	1×10^{-4}	4×10^{-6}
MOTOR CUE-AVG	2×10^{-4}	5×10^{-10}	9×10^{-3}	2×10^{-6}
GAMBLING PUNISH	6×10^{-8}	7×10^{-14}	10^{-14}	2×10^{-3}
GAMBLING REWARD	3×10^{-6}	9×10^{-13}	10^{-15}	3×10^{-3}

Supplemental Table 7: p-value from paired 2-tail t-test of R^2 scores between predicted and target task fMRI activation maps. The R2 scores are computed across 18 task contrasts whose target-repeat reliability R^2 is higher than 0 of 39 test subjects.

3. Supplemental Methods

3.1. fMRI preprocessing

Resting-state fMRI data from PIOP and IBC datasets were preprocessed using the FMRIPREP version stable [57], a Nipype [58] based tool. Each T1w (T1-weighted) volume was corrected for INU (intensity non-uniformity) using N4BiasFieldCorrection v2.1.0 [85] and skull-stripped using antsBrainExtraction.sh v2.1.0 (using the OASIS template). Brain surfaces were reconstructed using recon-all from FreeSurfer v6.0.1 [86], and the brain mask estimated previously was refined with a custom variation of the method to reconcile ANTs-derived and FreeSurfer-derived segmentations of the cortical gray-matter of Mindboggle [87]. Spatial normalization to the ICBM 152 Nonlinear Asymmetrical template version 2009c [88] was performed through nonlinear registration with the antsRegistration tool of ANTs v2.1.0 [89], using brain-extracted versions of both T1w volume and template. Brain tissue segmentation of cerebrospinal fluid (CSF), white-matter (WM) and gray-matter (GM) was performed on the brain-extracted T1w using fast [90] (FSL v5.0.9).

Functional data was slice time corrected using 3dTshift from AFNI v16.2.07 [91] and motion corrected using mcflirt (FSL v5.0.9 [92]). This was followed by co-registration to the corresponding T1w using boundary-based registration [93] with six degrees of freedom, using bbregister (FreeSurfer v6.0.1). Motion correcting transformations, BOLD-to-T1w transformation and T1w-to-template (MNI) warp were concatenated and applied in a single step using antsApplyTransforms (ANTs v2.1.0) using Lanczos interpolation.

Physiological noise regressors were extracted applying CompCor [94]. Principal components were estimated for the two CompCor variants: temporal (tCompCor) and anatomical (aCompCor). A mask to exclude signal with cortical origin was obtained by eroding the brain mask, ensuring it only contained subcortical structures. Six tCompCor components were then calculated including only the top 5% variable voxels within that subcortical mask. For aCompCor, six components were calculated within the intersection of the subcortical mask and the union of CSF and WM masks calculated in T1w space, after their projection to the native space of each functional run. Frame-wise displacement [15] was calculated for each functional run using the implementation of Nipype. Many internal operations of FMRIPREP use Nilearn [95], principally within the BOLD-processing workflow. For more details of the pipeline see https://fmriprep.readthedocs.io/en/stable/workflows.html.
